# A single-cell transcriptomic landscape characterizes the endocrine system aging in the mouse

**DOI:** 10.1093/procel/pwaf074

**Published:** 2025-09-01

**Authors:** Ran Wei, Zhehao Du, Jue Wang, Jinlong Bi, Wencong Lyu, Haochen Wang, Jianuo He, Fanju Meng, Lijun Zhang, Chao Zhang, Chen Zhang, Wei Tao

**Affiliations:** The State Key Laboratory of Membrane Biology, School of Life Sciences, Peking University, Beijing 100871, China; The State Key Laboratory of Membrane Biology, School of Life Sciences, Peking University, Beijing 100871, China; School of Basic Medical Sciences, Beijing Key Laboratory of Neural Regeneration and Repair, Beijing Laboratory of Oral Health, Capital Medical University, Beijing 100069, China; State Key Laboratory of Neurology and Oncology drug development, Nanjing 210000, China; Chinese Institute for Brain Research, Beijing 102206, China; The State Key Laboratory of Membrane Biology, School of Life Sciences, Peking University, Beijing 100871, China; The State Key Laboratory of Membrane Biology, School of Life Sciences, Peking University, Beijing 100871, China; The State Key Laboratory of Membrane Biology, School of Life Sciences, Peking University, Beijing 100871, China; The State Key Laboratory of Membrane Biology, School of Life Sciences, Peking University, Beijing 100871, China; The State Key Laboratory of Membrane Biology, School of Life Sciences, Peking University, Beijing 100871, China; The State Key Laboratory of Membrane Biology, School of Life Sciences, Peking University, Beijing 100871, China; State Key Laboratory of Genetic Resources and Evolution, Kunming Institute of Zoology, Chinese Academy of Sciences, Kunming 650201, China; School of Basic Medical Sciences, Beijing Key Laboratory of Neural Regeneration and Repair, Beijing Laboratory of Oral Health, Capital Medical University, Beijing 100069, China; State Key Laboratory of Neurology and Oncology drug development, Nanjing 210000, China; Chinese Institute for Brain Research, Beijing 102206, China; The State Key Laboratory of Membrane Biology, School of Life Sciences, Peking University, Beijing 100871, China

**Keywords:** single-cell RNA-seq, aging, endocrine system, immune infiltration, MHC-I, GZMK

## Abstract

The endocrine system is crucial for maintaining overall homeostasis. However, its cellular signatures have not been elucidated during aging. Here, we conducted the first-ever single-cell transcriptomic profiles from eight endocrine organs in young and aged mice, revealing the activation of cell-type-specific aging pathways, such as loss of proteostasis, genomic instability and reactive oxygen species (ROS). Among six sex-shared endocrine organs, aging severely impaired gene expression networks in functional endocrine cells, accompanied by enhanced immune infiltration and unfolded protein response (UPR). Mechanism investigations showed that expanded aging-associated exhausted T cells activated MHC-I–UPR axis across functional endocrine cells by releasing GZMK. The inhibition of GZMK receptors by small chemical molecules counteracted the UPR and senescence, suggesting the immune infiltration is a possible driver of endocrine aging. Machine learning identified CD59 as a novel aging feature in sex-shared functional endocrine cells. For two sex-specific endocrine organs, both aged ovaries and testes showed enhanced immune responses. Meanwhile, cell-type-specific aging-associated transcriptional changes revealed an enhanced ROS mainly in aged theca cells of ovaries, while aged spermatogonia in testes showed impaired DNA repair. This study provides a comprehensive analysis of endocrine system aging at single-cell resolution, offering profound insights into mechanisms of endocrine aging.

## Introduction

Aging is an intrinsic biological process characterized by the progressive deterioration of physiological functions and increased susceptibility to age-related diseases. The proposed mechanisms underlying aging include mitochondrial dysfunction, genomic instability, disrupted intercellular communication, chronic inflammation, loss of proteostasis, and cellular senescence (Amorim et al., 2022; Di Micco et al., 2021; Dodig et al., 2019; López-Otín et al., 2013, 2023; Strande et al., 2007). This complex process affects various systems within the body, including the endocrine system, which plays a pivotal role in hormone regulation and homeostasis maintenance (Cappola et al., 2023; van den Beld et al., 2018b).

The endocrine system, which includes the hypothalamus, pituitary gland, thyroid gland, adrenal gland, pineal gland, pancreatic islet, ovary, and testis undergoes complex changes during the aging process. These changes involve alterations in hormonal secretory patterns and modulation of feedback sensitivity (van den Beld et al., 2018). Aging is often accompanied by an increase in thyroid-stimulating hormone levels (van Heemst, 2024) and a gradual decline in growth hormone secretion (Cappola et al., 2023). Numerous studies have focused on investigating alterations in endocrine hormone levels within the bloodstream during the aging process and their effects on peripheral organs (Junnila et al., 2013; Karasek, 2004; van den Beld et al., 2018b; Wang et al., 2024b). In recent years, studies highlighting the molecular mechanisms of aging in individual endocrine organs or specific cell types, such as mouse hypothalamus, human pancreatic beta cells, and primate ovaries, have emerged (Augsornworawat et al., 2020; Hajdarovic et al., 2022; Wang et al., 2020). However, the global mechanism of endocrine system aging remains poorly understood, and comprehensive comparative studies regarding the aging of various endocrine organs are lacking.

Single-cell RNA sequencing (scRNA-seq) is widely used to analyze the transcriptomes of individual cells within a sample. It enables the simultaneous profiling of transcriptomes of thousands of cells, facilitating the construction of comprehensive cell atlases for various organs and organisms (Gao et al., 2020). In recent years, continuous advancements in single-cell transcriptomics have opened new possibilities for comprehensively investigating aging-related changes and mechanisms within multiple organs from the same system (Svensson et al., 2018; Uyar et al., 2020). Additionally, these studies reported common or organ-specific aging pathways by comparing different organs, expanding our understanding of the aging process in one system as a whole (Enge et al., 2017; Ma et al., 2020; Zhang et al., 2020).

In this study, we generated a comprehensive single-cell transcriptomic atlas of endocrine system aging. A total of 208,304 high-quality cells from eight endocrine organs were analyzed in young and aged mice, enabling us to explore transcriptomic changes during aging. Using this data, for sex-shared endocrine organs, we described changes in gene expressions and pathway alterations during aging and highlighted the immune microenvironment changes during endocrine aging, particularly the expansion in aging-associated CD8^+^ T cells, which are characterized by high expression GZMK. In addition, we revealed a new regulatory axis in aging, where GZMK activated the UPR by upregulating MHC-I in aged functional endocrine cells. Machine learning identified CD59 as a new feature of endocrine aging. For sex-specific endocrine organs, aging altered the shared and distinct pathways of the ovary and testis. Both gonads exhibited heightened immune responses during aging. Overall, our study provides a comprehensive single-cell transcriptomic atlas of aging in multiple endocrine organs, offering a valuable resource for future functional analysis of endocrine aging.

## Results

### Establishment of a single-cell transcriptomic atlas in six endocrine organs during aging

**Figure 1.**
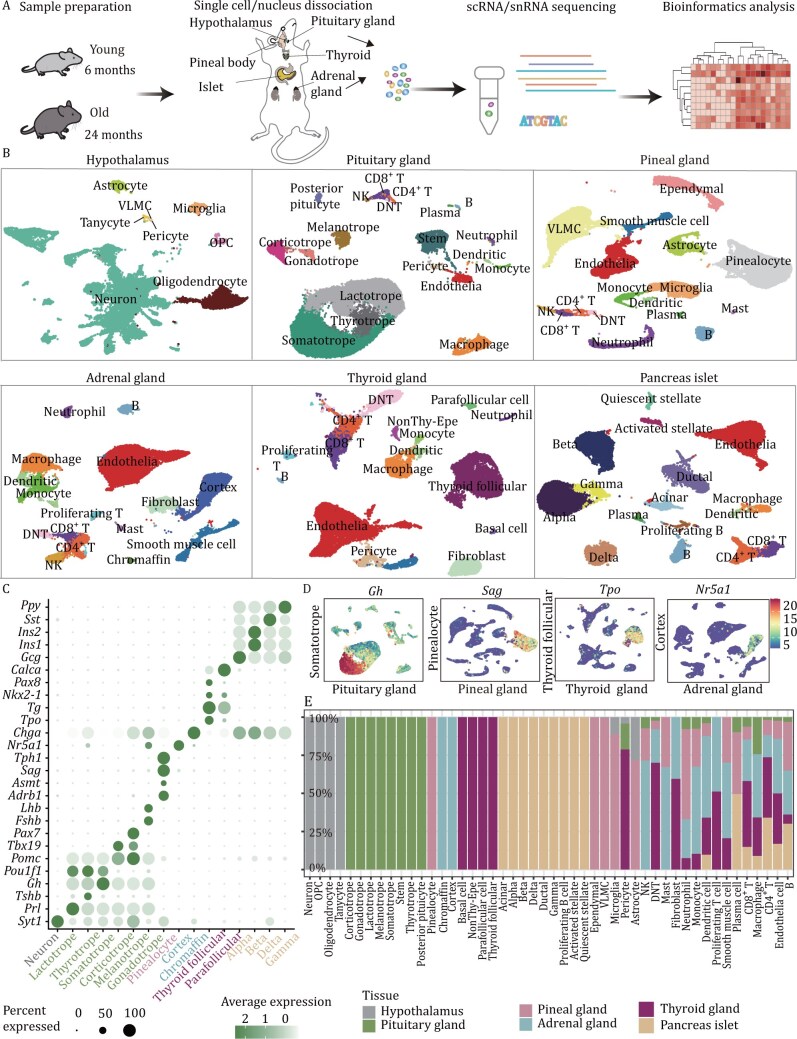
**Construction of a multiorgan single-cell transcriptomic atlas of endocrine aging in mice.** (A) Schematic diagram of the workflow for the establishment of a single-cell aging endocrine atlas. The full names of the cell types are as follows: NK, Natural killer cell; DNT, CD4^−^CD8^−^ T cell; OPC, oligodendrocyte precursor cell; VLMC, vascular leptomeningeal cells. (B) Uniform manifold approximation and projection (UMAP) plot of all 169,859 cells analyzed from six endocrine organs. (C) Dot plot showing the marker expression levels of functional endocrine cells responsible for hormone secretion in different endocrine organs. (D) UMAP plots of representative cell type-specific markers. *Gh*, pituitary somatotrophs; *Sag*, pinealocytes; *Tpo*, thyroid follicular cells; *Nr5a1*, adrenal cortex. The color coding indicates gene expression levels. (E) Bar chart illustrating organ heterogeneity across different cell types. Different colors represent different organs.

To elucidate the impact of aging on the endocrine system, we conducted single-cell/single-nucleus sequencing of representative endocrine organs from young (6-month-old) and aged (24-month-old) mice. For sex-shared endocrine organs, the pituitary glands, pineal glands, thyroid glands, adrenal glands, and pancreatic islets were dissected and processed for single-cell RNA sequencing (scRNA-seq). Additionally, from the hypothalamus, cell nuclei were extracted and subjected to single-nucleus RNA-seq (snRNA-seq) (Fig. 1A). After stringent quality control (Fig. S1A; Table S1), we obtained a total of 169,859 high-quality single cells/nuclei that constitute a comprehensive aging cell atlas of six sex-shared endocrine organs.

To assign cell types, we applied normalization, batch effect correction, dimensionality reduction, and clustering separately in each of the six distinct endocrine organs (Fig. 1B and S1B–D). We utilized canonical markers to accurately identify and annotate cell types within each organ (Fig. 1B–D; Table S2), such as *Gh*, a gene encoding growth hormone (Reh and Geffner, 2010), which distinguishes somatotrophs in the pituitary gland, and *Tpo*, a key enzyme involved in thyroid hormone synthesis(Doerge and Chang, 2002), which distinguishes thyroid follicular cells in the thyroid gland. In total, we identified 48 distinct cell types across the six endocrine organs (Fig. 1B and S1B–D), which is consistent with previous work (Fig. S2A–D). The high similarity of all the major cell types between our data and the public datasets demonstrated high data quality and accurate cell type annotations in our data (Fig. S2E). Among the cell types, 29 were found to be organ-specific, including 16 hormone secretion cell types: (i) lactotrophs, thyrotrophs, somatotrophs, corticotrophs, melanotrophs and gonadotrophs in the pituitary gland; (ii) neuropeptide-secreting neurons in the hypothalamus; (iii) pinealocytes in the pineal gland; (iv) adrenal cortex cells and chromaffin cells in the adrenal gland; (v) thyroid follicular cells and parafollicular cells in the thyroid gland; and (vi) alpha, beta, gamma and delta cells in the pancreatic islets (Fig. 1E). The remaining 19 cell types were distributed across multiple organs; for example, CD8^+^ T cells were found in the pituitary gland, pineal gland, adrenal gland, thyroid gland and pancreatic islet (Fig. 1E). In summary, we constructed a single-cell resolution endocrine aging atlas, providing a rich resource for researchers focused on endocrine function, aging, or aging-related diseases.

### Aging disrupts gene expression networks and dysregulates diverse pathways in a cell-type-specific manner within endocrine organs

**Figure 2.**
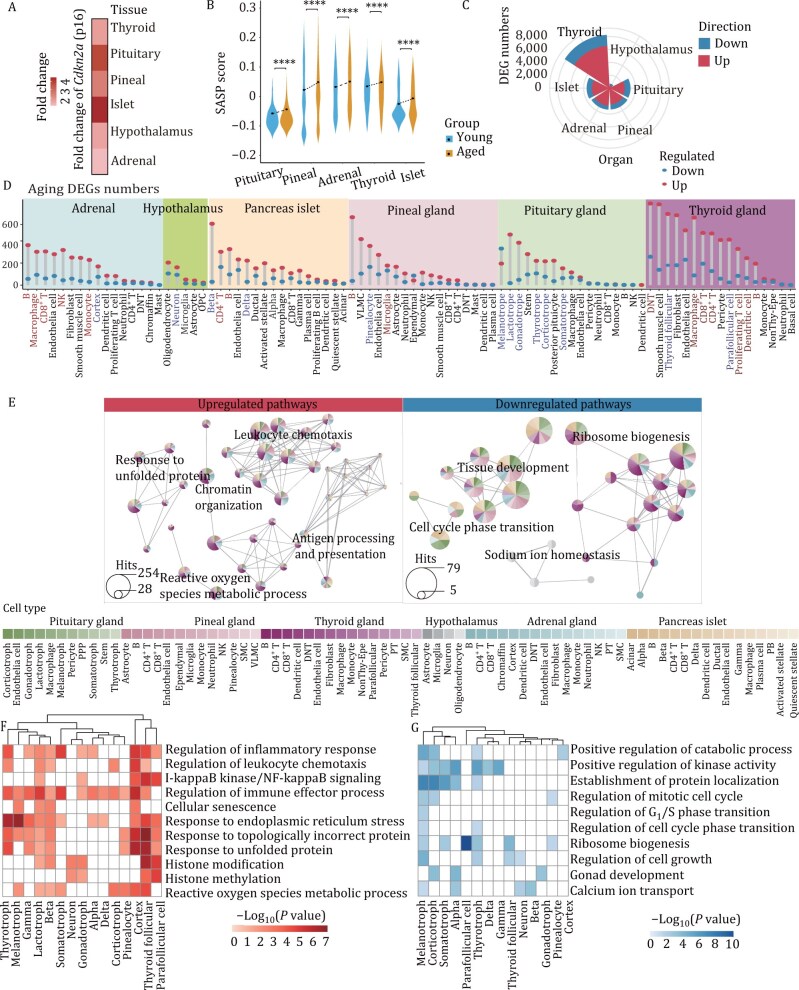
**Aging alters cell-type-specific pathways within endocrine organs.** (A) Heatmap demonstrating the fold change in the expression of *Cdkn2a* (p16) in six endocrine organs between aged and young mice. Red indicates fold changes greater than 0. (B) Violin and line plots showing SASP-related gene score changes in the pituitary, pineal, adrenal, thyroid gland and pancreatic islet of young and aged mice according to the Wilcoxon rank‒sum test. *P value ≤ 0.05; **P value ≤ 0.01; ***P value ≤ 0.001; ****P value ≤ 0.0001. The black dots represent the mean values for each group, and the dotted lines link the young and aged groups for each organ to visualize the aging-associated trends. (C) Rose diagram showing the number of aging-associated DEGs in various endocrine organs. (D) Lollipop plot illustrating aging-associated DEGs in different cell types of mice, grouped by tissue as indicated. Cell types with DEG numbers exceeding 200 are labeled, with immune cell names highlighted in red and functional endocrine cell names in blue. (E) Networks showing the enriched GO terms and pathways, including upregulated (left) and downregulated (right) DEGs for all cell types in 6 endocrine organs during aging. Each cell type is color-coded below for reference. (F and G) Heatmaps showing the representative aging-associated GO terms for genes with upregulated (F) and downregulated (G) expression in functional endocrine cells.

To assess the senescence status of different endocrine organs, we first examined the expression of the classic senescence marker *Cdkn2a* (p16) (Krishnamurthy et al., 2004). Consistent with a previous study regarding nonendocrine organs (Almanzar et al., 2020), we found that *Cdkn2a* expression was upregulated in six endocrine organs during aging (Fig. 2A). In addition, other senescence-related markers, such as the senescence-associated secretory phenotype (SASP) and SenMayo (Saul et al., 2022; Wang et al., 2024a), also showed significantly upregulated expression during aging (Figs. 2B and S3A). Moreover, previously reported aging-related genes (Hajdarovic et al., 2022; Vennekens et al., 2021) in specific cell types were also captured in our data (Fig. S3B). These results suggest that cellular senescence is accompanied by endocrine organ aging and may be a hallmark of endocrine aging.

Next, we focused on the molecular changes that occur during aging. First, we confirmed that the weighting of cell types across the six endocrine organs was balanced, ensuring that the analysis was not subject to potential biases caused by unequal weighting from different organs (Fig. S3C). In total, 5,261 genes were determined to be differentially expressed genes (DEGs) between aged and young mice. By analyzing aging-associated DEGs within each cell type, we observed high variations in the numbers of DEGs across different endocrine organs and cell types, indicating high aging-related heterogeneity in endocrine organs. At the organ level, the thyroid gland presented the greatest number of aging-associated DEGs (Fig. 2C; Table S3). At the cell type level, many cell types with a high number of aging-associated DEGs were found to be immune cells and functional endocrine cells, suggesting that these cells may undergo significant changes during aging (Fig. 2D). For example, CD8^+^ T cells exhibited a large number of DEGs in the adrenal and thyroid gland. Furthermore, these DEGs were enriched in inflammatory response and chromatin remodeling. We observed that two common DEGs in these two types of T cells, *Gzmk* and *Pdcd1* (T cell exhaustion markers), were upregulated with aging, suggesting a potential increase in exhausted T cells in the thyroid and adrenal glands (Fig. S3D and S3E). Functional endocrine cells involved in secreting hormones, such as adrenal cortex and thyroid follicular cells, also showed a relatively high number of DEGs. Notably, in the pituitary gland, the functional endocrine cells, including melanotrophs, lactotrophs, and gonadotrophs, exhibited the highest number of DEGs (Fig. 2D).

To further elucidate the biological functions associated with these molecular changes, we performed gene ontology (GO) enrichment analysis of aging-associated DEGs. Leukocyte chemotaxis, response to unfolded proteins, reactive oxygen species metabolic processes, and chromatin organization pathways were enhanced in various cell types (Fig. 2E). Consistently, *Jund*, *Cebpb*, and *Igkc*, which are key regulators of chronic inflammation(Behmoaras et al., 2008; Li et al., 2023b; Zahid et al., 2019), generally showed upregulated expression in multiple cell types (Fig. S3F). Notably, the enhanced inflammation-related pathways were shared among endothelial cells across various organs (Fig. S3G). As non-parenchymal components, immune cells exhibit enhanced inflammation-related responses across multiple endocrine organs, suggesting potential immune infiltration in endocrine organs during aging (Fig. S3H). Conversely, genes related to organ development, sodium ion homeostasis, the cell cycle phase transition, and the ribosome biogenesis pathway showed downregulated expression during endocrine aging (Figs. 2E and S3F).

In the endocrine system, parenchymal cells are the functional endocrine cells responsible for hormone secretion. As functional hormone-secreting cells in endocrine organs play central roles in diverse endocrine organs (Doerge and Chang, 2002), we next investigated aging-related pathways in functional endocrine cells. Their aging pathways include both cell type specific and shared mechanisms, highlighting the complexity of endocrine aging. Similar to the above results, we identified aging regulatory pathways, such as immune response, the endoplasmic reticulum stress (ER stress)-related unfolded protein response, chromatin remodeling, and reactive oxygen species metabolic processes, shared among multiple parenchymal cells of different endocrine organs, although the degree of activation varied across different cell types (Fig. 2F). For example, altered gene expressions related to the ER stress-related unfolded protein response was most pronounced in pituitary thyrotrophs, thyroid follicular cells, and the adrenal cortex and less pronounced but still enriched in pituitary lactotrophs and somatotrophs (Fig. 2F). Pathways associated with epigenetic alterations, including histone modifications, were enriched in aged thyroid follicular cells, parafollicular cells, gonadotrophs, neurons, pancreatic beta cells, and lactotrophs (Figs. 2F and S3I), which has been reported in other cell types (Wang et al., 2018; Yi and Kim, 2020). Similarly, chronic inflammation has been reported in multiple studies (Li et al., 2023a), and it is also increased in most functional endocrine cells except neurons as shared aging pathways (Fig. 2F). In addition, the upregulation of *Gadd45a* expression, an oxidative stress sensor gene (Hassan et al., 2024; Moskalev et al., 2012) associated with reactive oxygen metabolism processes, was observed in aged pinealocytes (Fig. S3J). In contrast, the mitotic cell cycle and ribosome biogenesis were downregulated in various functional endocrine cells (Fig. 2G). In summary, our findings elucidate the molecular basis of endocrine cell aging and reveal aging-related pathways in endocrine organs, providing potential targets for rejuvenation.

### The unfolded protein response and inflammation are activated in multiple aged functional endocrine cells

**Figure 3.**
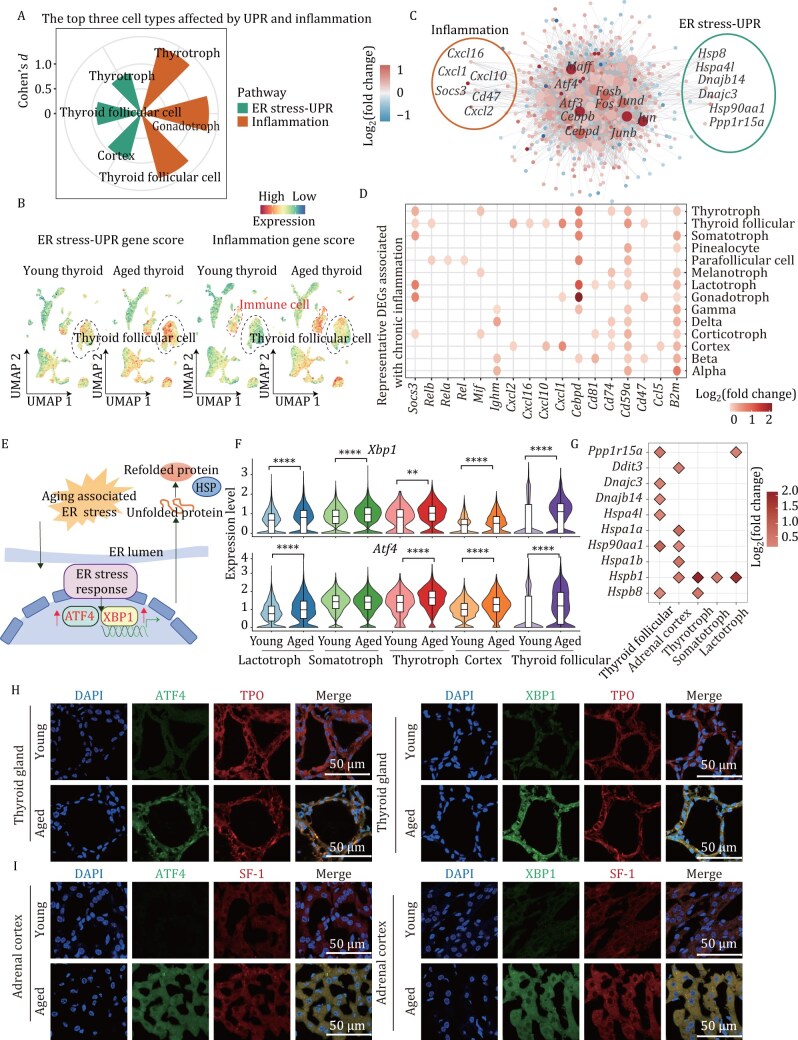
**The activation of the UPR and inflammation occurs in aged functional endocrine cells of the pituitary, thyroid, and adrenal glands.** (A) Rose diagram illustrating the top three functional cell types with the most significant changes in UPR and inflammation during aging. (B) UMAP plot showing ER stress associated with UPR scores and inflammation scores across young and aged in the thyroid glands. (C) Network visualization of transcription factor regulation of aging-associated DEGs in thyroid follicular cells. The internal nodes represent TFs, the circles with gray edges denote the downstream target DEGs of these TFs. The genes enriched in different aging pathways are indicated in the circular plots with different colored circles representing each category. (D) Dot plot showing representative DEGs related to inflammation in the indicated functional endocrine cells. (E) Schematic diagram of the enhanced UPR in functional endocrine cells during aging. (F) Violin plot showing that the UPR-related pathways mediated by *Atf4* and *Xbp1* show upregulated activity in aged thyroid follicular cells, the adrenal cortex, thyrotrophs, somatotrophs, and lactotrophs, according to the Wilcoxon rank‒sum test. *P value ≤ 0.05; **P value ≤ 0.01; ***P value ≤ 0.001; ****P value ≤ 0.0001. (G) Dot plot showing expression changes in protein refolding-related genes. (H and I) Representative immunofluorescence images of TPO and ATF4, TPO and XBP1 (H), SF-1 and ATF4, and SF-1 and XBP1 (I) double-positive cells across the indicated organs in the young and aged groups (*n* = 6). TPO can distinguish thyroid follicular cells, and SF-1 can distinguish cells of the adrenal cortex. Scale bars, 50 μm. The data are shown as the means ± SEMs.

The unfolded protein response (UPR) is an important pathway involved in maintaining protein homeostasis, and loss of proteostasis can lead to the accumulation of misfolded or aggregated proteins, which can have detrimental effects on endocrine cell function (Hetz et al., 2020). Notably, we identified that chronic inflammation and ER-stress-related UPR were significantly elevated and shared in multiple different functional endocrine cells (Fig. 2F). To further investigate the impact of chronic inflammation and ER stress on different functional endocrine cell types, we computed ER-stress and inflammation scores. Using Cohen’s *d* to measure the magnitude of the differences in UPR and inflammation scores between aged and young groups, we ranked the top three functional endocrine cells most affected by UPR and inflammation. Thyroid follicular cells, pituitary thyrotrophs, and gonadotrophs ranked as the top three for the increase in inflammation during aging, while thyroid follicular cells, adrenal cortex, and pituitary thyrotrophs ranked in the top three for the upregulation of UPR. These results suggest that these functional cell types undergo significant changes during aging (Figs. 3A, 3B, S4A and S4B). For example, the UPR and inflammation scores in thyroid follicular cells significantly increased during aging, although immune cells exhibited a more pronounced increase in inflammation (Fig. 3B). Additionally, adrenal cortex cells and pituitary lactotrophs, thyrotrophs, and somatotrophs exhibited varying degrees of enhanced UPR responses (Fig. S4B). To further explore the transcriptional regulatory networks underlying aging, we predicted the core transcription factors regulating aging-related DEGs in thyroid follicular cells and thyrotrophs, which exhibited significant increases both in UPR and inflammation-related pathways (Fig. 3C and S3K). The AP1 family, including *Atf4*, was predicted to target UPR and inflammation-related genes, suggesting it may play a key regulatory role (Fig. 3C and Fig. S3K).

We next studied the inflammation-related DEGs in functional endocrine cells during aging. The chemokine CXCL family, including *Cxcl2*, *Cxcl16*, *Cxcl10*, and *Cxcl1*, was upregulated in various functional endocrine cells with aging (Fig. 3D). Additionally, β2-microglobulin (B2M), a component of major histocompatibility complex class I (MHC I) molecules and a known pro-aging factor(Smith et al., 2015), was also upregulated in multiple functional endocrine cells (Fig. 3D). To gain further insight into UPR-related gene expression alterations across the endocrine system, we focused on the key transcriptional factors *Xbp1* and *Atf4* (Harding et al., 1999; Lu et al., 2004; Winnay et al., 2010), which play regulatory roles in the UPR pathway (Fig. 3E). We observed the upregulation of *Atf4 and Xbp1* expression in various functional endocrine cells (Fig. 3F), which was further validated by immunofluorescence staining of XBP1 and ATF4 in aged thyroid follicular cells (distinguished by TPO expression), the adrenal cortex (distinguished by SF-1 expression), pituitary lactotrophs, somatotrophs and thyrotrophs (commonly distinguished by PIT1 expression) (Figs. 3H, 3I, S4C and S4E).

To further confirm the activation of the UPR pathway in these functional endocrine cells, we next investigated the downstream genes of the UPR, including heat shock proteins (HSPs), which assist in protein folding. We detected significantly increased expression of genes encoding HSPs in aged thyroid follicular cells, the adrenal cortex, and pituitary lactotrophs, somatotrophs, and thyrotrophs (Fig. 3G). For example, the genes encoding 70-kDa heat shock proteins (HSP70s) (Chen et al., 2023), including *Hspa1b*, *Hspa1a*, *and Hspa4l,* which are molecular chaperones involved in a variety of cellular protein folding processes(Rosenzweig et al., 2019), presented increased expression in thyroid follicular cells and the adrenal cortex. Subsequent immunofluorescence staining confirmed the upregulation of HSP70 expression in the adrenal and thyroid gland during aging (Fig. S4D and S4E). In summary, the UPR and downstream responses are enhanced in aged functional endocrine cells of the thyroid, adrenal, and pituitary gland, indicating that the UPR pathway is widely activated during endocrine aging.

### Exhausted GZMK^+^CD8^+^ T cells accumulate in endocrine aging

**Figure 4.**
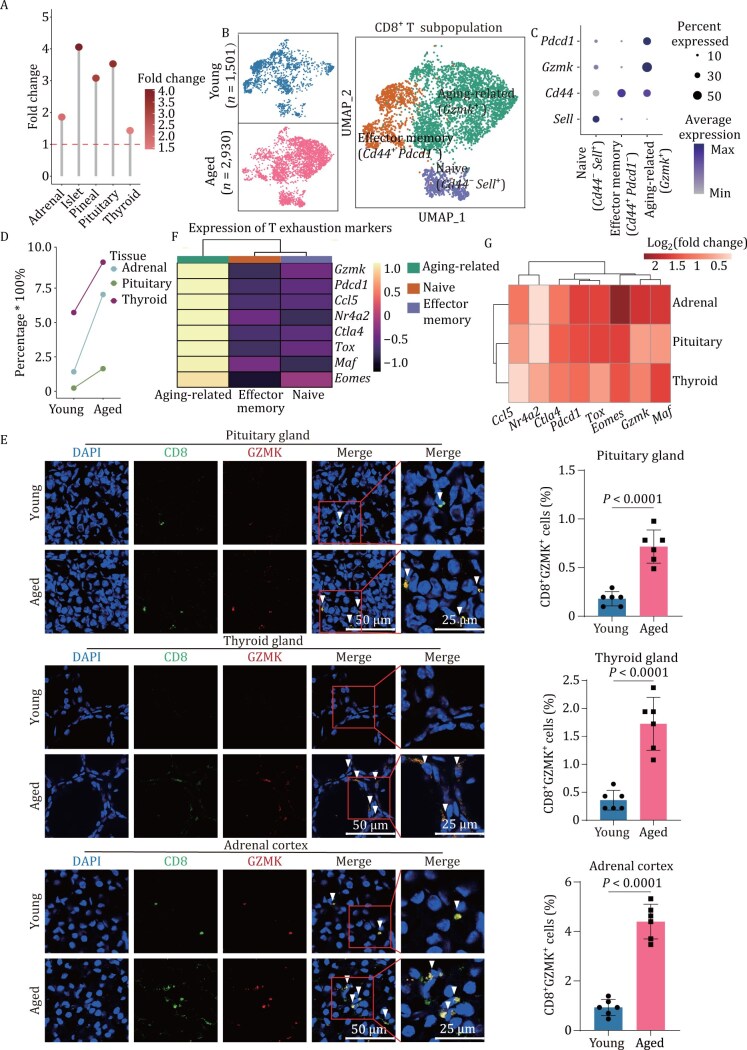
**Aging-associated exhausted GZMK** ^**+**^ **CD8** ^**+**^  **T cells expand in aged pituitary, thyroid and adrenal glands.** (A) Dot plot illustrating the fold changes (aged/young) in the proportions of immune cells across endocrine organs. All the fold changes are greater than 1. The color key represents the magnitude of the fold change. (B) UMAP showing subpopulations of CD8^+^ T cells in the pituitary, thyroid, and adrenal glands. The left panel shows the distribution of CD8^+^ T cells in the young and aged groups. The right panel shows all cells from young and aged mice, which are colored according to the subpopulation identified. (C) Dot plot showing the marker gene expression levels of CD8^+^ T cell subpopulations within the indicated organs. (D) Line plot showing changes in the proportions of aging-associated GZMK^+^CD8^+^ T cells within the indicated organs during aging. (E) Representative immunofluorescence images (left) and quantification (right) of CD8^+^GZMK^+^ cells across the indicated organs in the young and aged groups (*n* = 6). (F) Heatmap demonstrating the proportion of GZMK^+^CD8^+^ T cells expressing T cell exhaustion-related genes in contrast to other subpopulations. (G) Heatmap illustrating the upregulation of T cell exhaustion-related genes in GZMK^+^CD8^+^ T cells during aging.

We next investigated the immune microenvironment of aged endocrine organs. The proportion of immune cells increased with age in most endocrine organs, suggesting an increase in immune infiltration and chronic inflammation in endocrine organs (Fig. 4A). Interestingly, the percentage of CD8^+^ T cells increased with age (Fig. S5A). Specifically, a subpopulation of CD8^+^ T cells that highly expressed granzyme K (GZMK^+^CD8^+^ T cells) was significantly expanded in endocrine organs, including the adrenal, pituitary, and thyroid gland (Fig. 4B–D). In addition, the immunofluorescence staining results supported the expansion of GZMK^+^CD8^+^ T cells during endocrine aging (Fig. 4E). Together, these results suggest the activation of a particular CD8^+^ T cell subpopulation distinguished by GZMK expression in aged endocrine organs, potentially shaping the immune microenvironment of endocrine organs.

To understand the function of GZMK^+^CD8^+^ T cells, we explored the molecular features of these cells and found that they highly expressed markers of T cell exhaustion, including the inhibitory receptors *Pdcd1* and *Ctla4*, as well as the related transcription factors *Tox*, *Nr4a2*, *Maf,* and *Eomes* (Blank et al., 2019; Wherry and Kurachi, 2015) (Fig. 4F). These results illustrate that the GZMK^+^ CD8^+^ T cells subpopulation shares the same features as exhausted T cells. GZMK has been reported to independently exacerbate the SASP (Mogilenko et al., 2021), so this subpopulation expressing *Gzmk* and *Ccl5* has proinflammatory potential (Fig. 4F). Furthermore, T cell exhaustion and proinflammatory features in GZMK^+^ CD8^+^ T cells increased with age in multiple organs (Fig. 4G and Fig. S5B). In summary, we revealed that aging-associated GZMK^+^CD8^+^ T cell expansion is associated with T cell exhaustion and proinflammatory features during endocrine organ aging.

### Functional endocrine cells highly express chemokines and MHC-I

**Figure 5.**
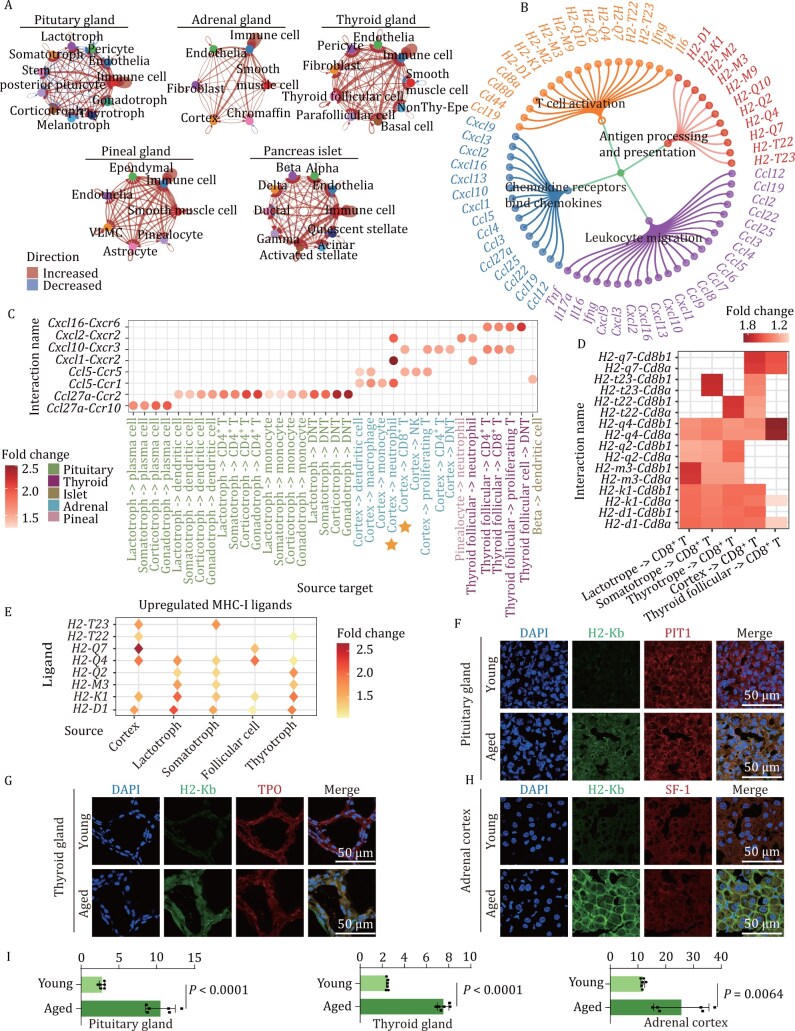
**Endocrine aging enhances immune cell recruitment-related pathways and MHC-I antigen presentation.** (A) Networks showing the changes in cell–cell interactions across cell types within the indicated organs. The color indicates the direction of change, and the line thickness indicates the change in interaction counts. (B) Network demonstrating the enriched pathways of cell‒cell interaction-related genes with upregulated expression. (C) Dot plot showing the fold changes (aged/young) in the probability of ligand‒receptor interaction in the CXCL and CCL signaling pathways. The vertical axis represents ligand‒receptor interactions, and the horizontal axis represents the source cell type and target cell type. The interactions mentioned are highlighted by yellow stars. (D) Heatmaps depicting the fold changes in the communication probability of ligand‒receptor interactions in MHC-I-related signaling pathways. (E) Dot plot illustrating the expression changes in ligands of the MHC-I signaling pathway across functional endocrine cell types. The color indicates the expression fold change of the corresponding ligand gene between the aged and young samples. (F‒H) Representative immunofluorescence images of H2-Kb^+^ cells coexpressing PIT1 (F), TPO (G) and SF-1 (H) across the indicated organs in the young and aged groups (*n* = 6). (I) Quantification of H2-Kb coexpression with PIT1, TPO, and SF-1 across the indicated organs in the young and aged groups (*n* = 6).

Altered intercellular communication is one of the hallmarks of aging (López-Otín et al., 2023), so we next focused on altered intercellular communication in endocrine organs. We analyzed cellular communication separately in different endocrine organs. The number of ligand‒receptor interactions increased in endocrine organs during aging, especially interactions associated with immune cells (Fig. 5A). Moreover, more than half of the upregulated ligand‒receptor interactions were associated with immune cells in each endocrine organ (Fig. S6A and S6B), indicating dramatic changes in immune-related cellular communication. GO and pathway enrichment analysis of immune-related cellular communication revealed increased chemotaxis and leukocyte migration, which is consistent with the findings of previous studies in other organs (Song et al., 2023; Yousefzadeh et al., 2018) (Fig. 5B). In particular, the expression of the chemokines CCL and CXCL was increased in functional endocrine cells (Fig. S6D). CCL and CXCL interactions between functional endocrine cells and immune cells were strengthened with aging (Fig. 5C), suggesting that functional endocrine cells secrete chemokines to communicate with and recruit immune cells during aging. For example, *Ccl5* expression was upregulated in the adrenal cortex, which was able to recruit CD8^+^ T cells. Additionally, aged adrenal cortex cells exhibited increased expression of *Cxcl1* and *Cxcl2,* thereby attracting neutrophils, which is consistent with the increased proportion of neutrophils in aged adrenal cortex cells (Figs. 5C and S6C).

Because T cell exhaustion occurs when T cells are chronically exposed to high quantities of antigens (Wherry and Kurachi, 2015) and an increase in T cell expression of exhaustion-related markers is observed in multiple endocrine organs during aging, we speculated that T cells may engage in cell‒cell interactions with surrounding cells during aging. By comparing the cell communication patterns between young and aged cells in each endocrine organ, we found a significant increase in the interaction between functional endocrine cells and CD8^+^ T cells through MHC-I and the *Cd8a*/*Cd8b1* ligand‒receptor pair (Figs. 5D and S6D–F). Specifically, we observed an increase in MHC-I ligand gene expression in functional endocrine cells such as those in the adrenal cortex, thyroid follicular cells, lactotrophs, somatotrophs, and thyrotrophs in the pituitary gland with age (Fig. 5E). Consistently, our immunofluorescence staining results also revealed increased numbers of MHC Class I (H2-Kb)-positive cells in aged functional endocrine cells (Fig. 5F–I). These results indicate that enhancement of the MHC-I pathway may play a crucial role in the immune response and the functional impairment of CD8^+^ T cells during aging in the endocrine system.

In summary, the immune microenvironment of multiple endocrine tissues underwent significant changes, particularly with functional endocrine cells expressing high levels of immune chemokines, suggesting enhanced recruitment of immune cells through increased chemokine secretion during aging. MHC-I ligand gene expressions in functional endocrine cells increased during aging, suggesting CD8^+^ T cells may exhibit increased interactions with multiple functional endocrine cells via MHC-I antigen presentation in the thyroid, adrenal, and pituitary gland. These results suggested aging reshaped the immune microenvironment through MHC-I antigen presentation and immune cell recruitment pathways.

### GZMK induces UPR and senescence via MHC-I activation, while inhibiting the GZMK receptors counteracts UPR and senescence

**Figure 6.**
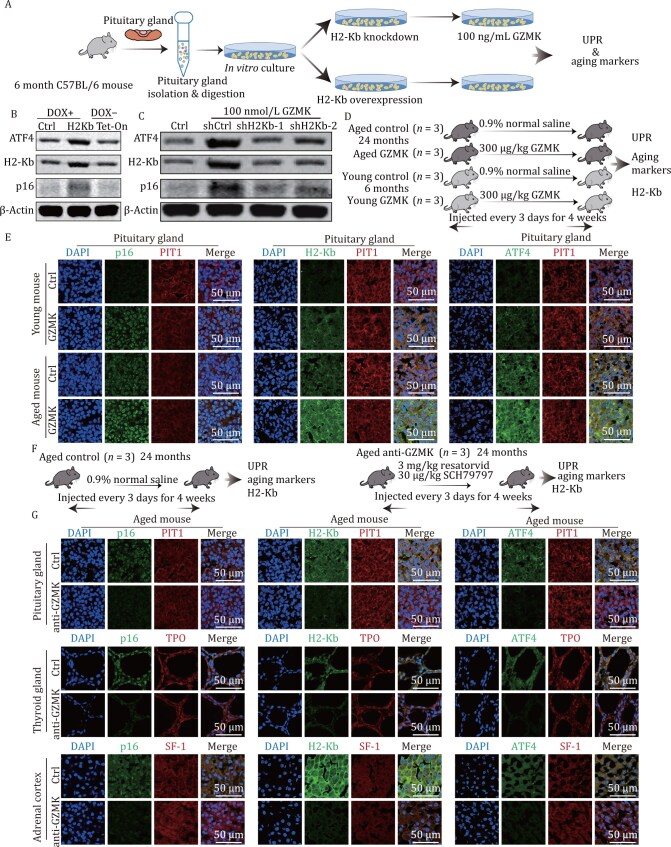
**Inhibiting GZMK receptors by small chemical molecules antagonizes the UPR and senescence.** (A) Schematic diagram of the functional evaluation of aged pituitary cells treated with different aging-associated factors. (B) Western blot analysis of the protein expression of H2-Kb and ATF4, key transcription factors of the UPR, following the overexpression of H2-Kb in pituitary cells. DOX, doxycycline. The Tet-On system is a tetracycline-inducible system that is transfected into cells to allow the activation of H2-Kb induced by DOX. *In vitro* cultured mouse pituitary cells showed significant upregulation of the expression of ATF4 and p16 after DOX-induced overexpression of H2Kb. (C) Western blot analysis of the protein expression of H2-Kb and ATF4 in pituitary cells treated with 100 ng/mL GZMK for knocking down H2-Kb expression and control group. (D) Schematic diagram showing the experimental design to investigate the relationship between GZMK and MHC-I, the UPR, and senescence markers in individual mice. The experimental mice were divided into four groups, namely, the aged control group, the aged GZMK-injected group, the young control group, and the young GZMK injection group, with a total of three biological replicates in each group. We used 6-month-old male mice for the young group and 24-month-old male mice for the aged group. Both the aged and young GZMK-injected groups were injected with granzyme K every 3 days for a total of 4 weeks, whereas in the control group, young and aged mice were injected with equal volumes of saline. (E) Representative immunofluorescence images of PIT1^+^ cells coexpressing p16, H2-Kb and ATF4 across the indicated pituitary glands in the control and treatment groups (*n* = 3). Ctrl, Control. (F) Schematic diagram of the experimental design for anti-GZMK treatment in mouse models. (G) Representative immunofluorescence images of PIT1, TPO, or SF-1 coexpression with p16, H2-Kb, and ATF4 across the indicated tissues in the control and anti-GZMK treatment groups (*n* = 3). Ctrl, Control.

We observed the expansion of GZMK^+^CD8^+^ T cells in multiple endocrine organs and the upregulation of MHC-I expression in aged endocrine-related cells. The role of GZMK as an inflammatory factor in activating immune responses has been widely reported (Joeckel et al., 2011; Jonsson et al., 2022; Turner et al., 2019). Previous studies have suggested that inflammatory factors function in MHC-I expression and the UPR (Coomans de Brachène et al., 2018; Fang et al., 2021; Jiang et al., 2022; Onabajo et al., 2021), which were also upregulated in our data (Figs. 3F and 5E). Therefore, we further explored whether GZMK could regulate MHC-I and the UPR and thereby contribute to endocrine aging. To this end, we isolated and cultured mouse pituitary cells *in vitro* and then established expression of H2-Kb, an MHC-I heavy chain, and overexpressed or knocked down H2-Kb expression in cell lines via a gene editing approach. By directly adding GZMK to the media, we then evaluated the expression of key transcription factors involved in the UPR and senescence markers (Fig. 6A).

After H2-kb was overexpressed, the expression of ATF4 (a key transcription factor of the UPR (Han et al., 2013; Hetz et al., 2020)) was significantly elevated in pituitary cells, indicating that H2-Kb could activate the UPR (Figs. 6B and S7A). In addition, the protein expression levels of H2-Kb and ATF4 were upregulated by treatment with GZMK. More importantly, the upregulation of ATF4 was abolished by knocking down of H2-Kb (Figs. 6C and S7B), suggesting that GZMK can regulate UPR through H2-Kb. Furthermore, we investigated the expression of the senescence marker p16 and found that GZMK could upregulate p16 (Figs. 6C and S7B), suggesting that GZMK plays a central role in endocrine organ aging. In summary, GZMK treatment can activate the UPR by increasing H2-Kb expression and inducing cellular senescence in endocrine organs.

We next investigated the function of GZMK *in vivo.* Injection of GZMK in both young and aged mice increased the expression of the senescence marker p16 in thyroid follicular cells, the adrenal cortex, and pituitary functional endocrine cells, including lactotrophs, somatotrophs, and thyrotrophs (Figs. 6D, 6E, S8A and S8B), demonstrating the regulatory role of GZMK in cellular senescence. Moreover, expression of MHC-I heavy chain H2-Kb and key factors involved in the UPR, ATF4 and XBP1, also increased as a result of GZMK injection (Figs. 6E, S8C, S8D, and S9A–D).

In contrast, injection of small molecules resatorvid and SCH79797, which are known inhibitors of the downstream receptors of GZMK, TLR4 and PAR1, respectively (Bouwman et al., 2021; Di Serio et al., 2007; Matsunaga et al., 2011; Sharma et al., 2016; Wensink et al., 2014), decreased p16, MHC-I, ATF4 and XBP1 expression in aged mice (Figs. 6F, 6G, S9A and S9B), implying that blocking the downstream pathway of GZMK signaling is capable of offsetting the UPR and delaying endocrine organ aging *in vivo*.

To verify the effects of anti-GZMK treatment on behavioral phenotypes and endocrine function in aged mice, we examined changes in key hormones secreted by the pituitary gland, thyroid gland, and adrenal cortex before and after treatment, as well as alterations in classical behavioral parameters and blood senescence-associated secretory phenotype (SASP) levels. The results showed that anti-GZMK treatment reduced the aging-induced elevations in blood thyroid-stimulating hormone (TSH), adrenocorticotropic hormone (ACTH), and glucocorticoid (GC) levels, whereas GZMK injection further increased these hormone levels (Fig. S10A). Additionally, anti-GZMK treatment upregulated growth hormone (GH) and triiodothyronine (T3) levels, which were reduced due to aging, while GZMK injection further suppressed them (Fig. S10A). Regarding behavioral phenotypes, anti-GZMK treatment improved the performance of aged mice in the open-field test, Y-maze test, and rotarod test, demonstrating its beneficial effects on locomotor and cognitive functions (Fig. S10B). Finally, we assessed changes in multiple SASP factors in the blood and found that levels of IL-1, IL-6, IL-10, TNF-α, and CXCL5 were significantly reduced after treatment. Collectively, these findings collectively demonstrate the intervention effects of anti-GZMK treatment on aging-related phenotypic and endocrine aging in mice (Fig. S10C).

Overall, our results demonstrate that GZMK functions as a direct aging factor by activating MHC-I to increase the UPR and promote cellular senescence; the inhibition of downstream receptors of GZMK antagonizes the UPR and endocrine aging.

### Machine learning identifies CD59 as a new feature of most functional endocrine cells during aging

**Figure 7.**
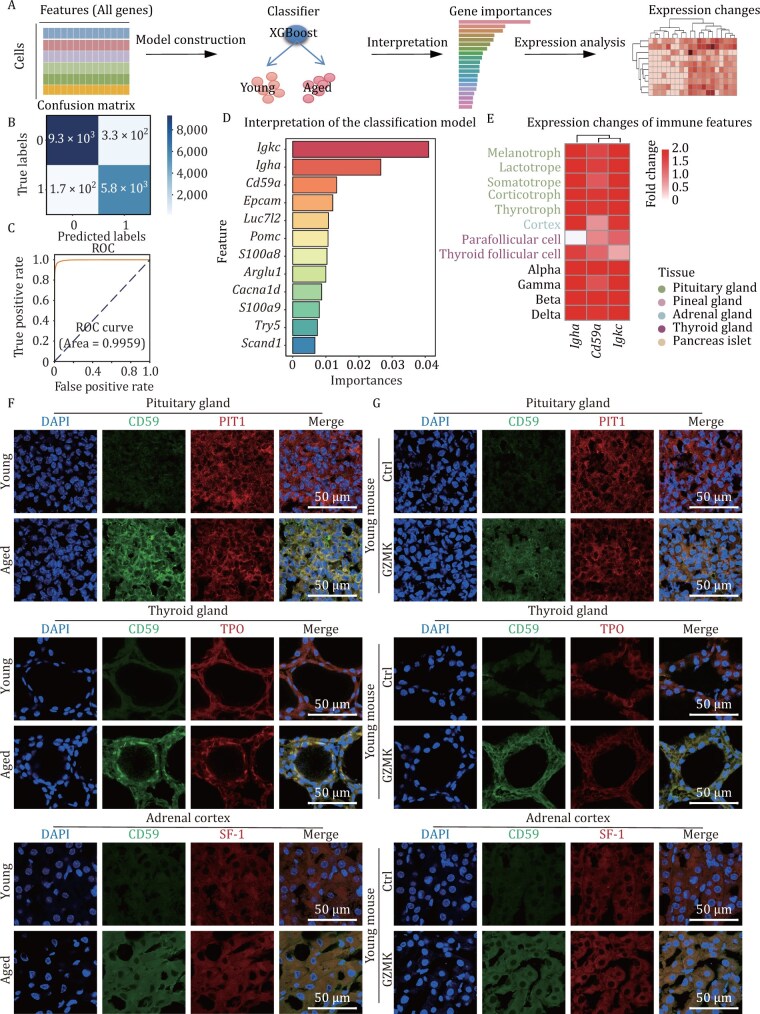
**Machine learning identifies CD59 as a vital feature of endocrine aging.** (A) Schematic illustrating the process of discovering features associated with the aging of functional endocrine cells using machine learning. (B) Confusion matrix depicting the accuracy of the XGBoost models predicting aged and young functional endocrine cells. (C) ROC curves of XGBoost models for predicting aged and young functional endocrine cells. (D) Bar chart showing the top features (ranked by importance) in the XGBoost models measured by normalized gain. (E) Heatmap showing the fold change of expression levels of *Igkc, Igha*, and *Cd59a* in young and aged functional endocrine cells. (F) Representative immunofluorescence images of CD59^+^ cells coexpressing PIT1, TPO, and SF-1 across the indicated organs in the young and aged groups (*n* = 6). (G) Representative immunofluorescence images of CD59^+^ cells coexpressing PIT1, TPO, and SF-1 across the indicated organs in the control and the young GZMK injection group groups (*n* = 3).

To identify the common characteristics of aging in sex-shared functional endocrine cells, we leveraged the interpretability of machine learning methods to mitigate the impacts of thresholds of differential gene expression and elucidate the shared features underlying the aging process in functional endocrine cells. We trained XGBoost models to classify cells as young or aged functional endocrine cells. Initially, all genes were inputted as features to train the XGBoost classifier. Then, we interpreted the results to indentify representative features (Fig. 7A). The classifiers achieved a high accuracy of 99.59% on test data set. Further validation was performed using confusion matrix and the receiver operating characteristic (ROC) curves, which confirmed the outstanding performance (Fig. 7B and 7C). Next, we employed the normalized gain from the XGBoost model to interpret the classification results. Finally, we identified the shared features by analyzing the changes in expression levels among various functional endocrine cells with aging.

Notably, three immune-related features, *Igkc*, *Igha*, and *Cd59a,* emerged as top important features in predicting aging in functional endocrine cells (Fig. 7D). By analyzing the changes in expression levels during aging, we found that these immune-related genes, *Cd59a, Igkc,* or *Igha,* exhibited upregulated expression with aging in most functional endocrine cells, suggesting the common emergence of an immune-like phenotype in aged functional endocrine cells (Fig. 7E). It is not surprising that *Igkc* and *Igha* have been identified as an aging characteristic of endocrine organs, as previous research has shown that it is important features of aging in endocrine organs (Li et al., 2023b). We further validated the upregulation of CD59 expression during aging in thyroid follicular cells, the adrenal cortex, pituitary lactotrophs, somatotrophs and thyrotrophs via immunofluorescence (Fig. 7F). Moreover, injecting GZMK into young or aged mice enhanced CD59 (encoded by *Cd59a* gene) expression, while the injection of a GZMK inhibitor reduces CD59 expression in aged mice (Figs. 7G and S11A–F). These results suggest that CD59 serves as a new feature of endocrine aging, and it may have a potential link with GZMK in endocrine aging.

In summary, we established a machine learning model for predicting cellular aging and, by interpreting its features, discovered the upregulation of immune-related genes such as *Cd59a*, *Igkc*, and *Igha* in aged functional endocrine cells. This indicates the immune-like transformation of functional endocrine cells with aging and suggests the potential of CD59 as a new feature for endocrine-immune aging

in most functional endocrine cells.

### In addition to immune infiltration the ovaries and testes undergo cell-type-specific aging-associated transcriptional changes

**Figure 8.**
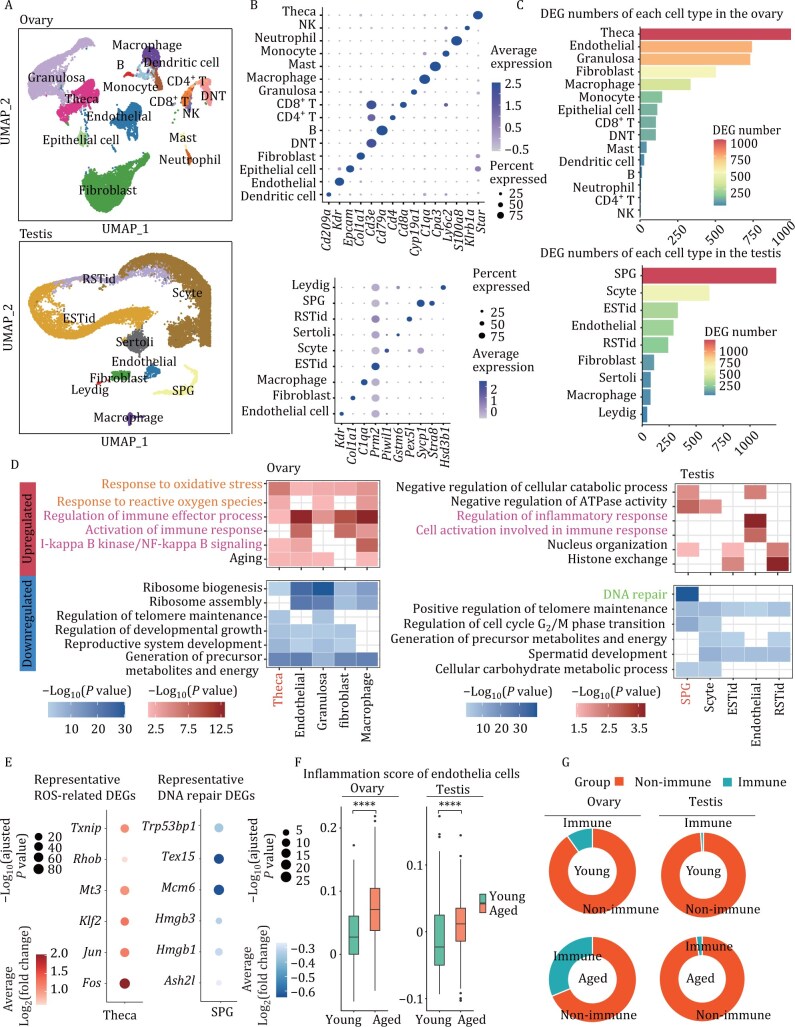
**Sex-specific endocrine organs exhibited distinct aging pathways.** (A) UMAP plot of cells in testis and ovary during aging. (B) Dot plot showing the marker expression levels of cell types in different endocrine organs. (C) Bar plot showing the number of aging-associated DEGs in various cell types in the ovary and testis. (D) Representative GO terms enriched in aging-associated DEGs in cell types in the ovary and the testis (*P* value < 0.05). (E) Dot plot showing representative differentially expressed genes associated with oxidative stress that are upregulated in the theca cells and DNA repair-related genes that are downregulated in the SPG cells. Red represents significantly upregulated genes, while blue represents significantly downregulated genes. (F) Box plot showing the inflammation scores of endothelial cells in the ovaries and testes for the young and aged groups, according to the Wilcoxon rank‒sum test. *P value ≤ 0.05; **P value ≤ 0.01; ***P value ≤ 0.001; ****P value ≤ 0.0001. (G) Pie chart showing the proportion of immune cells in the indicated group.

We previously explored the aging pathways in the shared endocrine organs of both females and males. To investigate the aging pathways in sex-specific endocrine organs, we next examined the aging transcriptomes of the female-specific ovary and male-specific testes. We conducted single-cell transcriptome analysis of the ovaries and testes in both young and aged mice, identifying the major cell types in the gonads (Fig. 8A and 8B). We then performed differential gene analysis for each cell type between the young and aged groups, identifying aging-associated DEGs in the gonads. In the ovary, Theca cells, which are involved in steroid hormone synthesis, exhibited the highest number of DEGs. In the testes, spermatogonia (SPG) cells showed the highest number of aging-associated DEGs, suggesting that these two cell types undergo significant changes during aging (Fig. 8C).

To further explore the shared and sex-specific aging responses in young and aged gonads, we conducted gene ontology (GO) analysis on the aging-associated DEGs from each group. In the ovary, theca cells upregulated responses to reactive oxygen species (ROS), while spermatogonia (SPG) in the testes downregulated DNA repair pathways during aging (Fig. 8D and 8E), which were also validated in public datasets (Fig. S12A and S12B). Both the ovary and testes exhibited enhanced immune-related responses and downregulated pathways associated with development and metabolism (Fig. 8D). Specifically, endothelial cells, a cell type common to both organs, showed increased inflammation with aging, suggesting a shared aging pathway between the two organs (Fig. 8D and 8F). We analyzed common up- and downregulated DEGs between public datasets and our data and performed pathway enrichment analysis. The analysis validated an enhanced ROS response in ovarian theca cells, reduced DNA repair in testicular SPGs, and upregulated immune pathways in endothelial cells of both the ovaries and testes (Fig. S12C and S12D). Furthermore, to investigate changes in the immune microenvironment of the gonads, we calculated the proportion of immune cells in the ovaries and testes from both young and aged groups. We found that immune cell proportions increased in both the ovaries and testes with aging, while the proportion of immune cells was higher in the ovaries (Fig. 8G and S12E), suggesting enhanced immune infiltration in both gonads with age. This increase was also observed in the proportion of immune cells in the aged ovaries and testes in public datasets.

In summary, the ovaries and testes exhibited both shared and distinct aging pathways. Theca cells in the ovaries showed an enhanced ROS response, while SPG in the testes exhibited reduced DNA repair. Both organs also displayed an increased immune response.

## Discussion

The endocrine system plays a crucial role in regulating various physiological processes and maintaining overall health and homeostasis in the body (Chahal and Drake, 2007). An increasing number of studies have investigated the aging of endocrine organs, including that of the pancreatic islets, ovaries, and hypothalamus, among others (Enge et al., 2017; Hajdarovic et al., 2022; Wang et al., 2020). However, these studies usually focus on a single organ, and a comprehensive study of endocrine system aging investigating the molecular mechanisms underlying the aging process in different endocrine organs and cell types is lacking. By establishing the first single-cell aging atlas of various endocrine organs, we identified several common molecular changes and potential regulatory pathways involved in endocrine aging. Thus, our work provides a valuable resource for understanding the molecular mechanisms of endocrine aging, enabling cross-organ and cross-species comparisons and offering insights for precise interventions targeting aging processes.

Although functional endocrine cells play crucial roles in hormone secretion as parenchymal components, previous researches have focused primarily on changes in hormone secretion during aging (Hertoghe, 2005; Junnila et al., 2013), and limited investigations and comparative analyses of the aging pathways in these cells have been undertaken. By leveraging our single-cell endocrine aging atlas, we confirmed the upregulation of activity of well-known aging pathways, such as those related to chronic inflammation, the oxidative stress response, protein homeostasis, cellular senescence, and histone modifications across different functional endocrine cell types during aging. Specifically, we found enhanced immune responses in various functional endocrine cells across different endocrine organs, along with upregulated UPR in functional endocrine cells of the thyroid, adrenal, and pituitary gland. These findings suggest a conserved role of immune activation and cellular stress responses in aging endocrine tissues. Furthermore, as non-parenchymal components, immune cells exhibited an enhanced immune response. Regarding the interplay between immune cells and functional endocrine cells, the CCL and CXCL pathways, along with the MHC-I pathway, are upregulated. We also established novel functional connections among aging pathways. Specifically, our research revealed the relationship between MHC-I expression and the UPR, leading to the identification of a novel aging pathway, the MHC-I–UPR aging regulatory axis, shared in functional endocrine cells of the pituitary, thyroid, and adrenal gland. It has been reported that MHC-I is involved in UPR-mediated pathways in other cell types and biological processes (Fréret et al., 2013), which supports our results. The MHC-I–UPR aging-related regulatory axis not only affects endocrine aging but also may be conserved across other organs and species.

While immune infiltration during aging has received considerable attention in recent years, the relationship between endocrine aging and immune aging remains largely unexplored (Brunet et al., 2022; Ma et al., 2022). In line with the senescent T cell exhaustion observed in the spleen, lungs, peritoneum, and kidneys (Mogilenko et al., 2021), we identified widespread expansion of GZMK^+^CD8^+^ exhausted T cells in the aged endocrine system, suggesting a potential role for these cells as immunological hallmarks of aging. Furthermore, we discovered that GZMK activated the UPR by enhancing MHC-I expression in the pituitary, thyroid, and adrenal gland, indicating that inflammation may promote endocrine aging. Moreover, the inhibition of downstream GZMK receptors via administration of the small molecules resatorvid and SCH79797 in individual mice counteracted cellular senescence and the UPR, providing a potential target for rejuvenation and healthy aging.

Immune infiltration is currently recognized as a key factor in aging, yet how it induces downstream aging pathways remains unclear. Our findings revealed enhanced immune infiltration within the endocrine system. Within this immune microenvironment, the immune factor GZMK induced the high expression of MHC-I in functional endocrine cells, leading to the loss of protein homeostasis and cellular senescence. We have demonstrated that this axis exists in functional endocrine cells, but the aging regulatory axis of other cell types is still unclear. Considering that immune infiltration is common across all endocrine organs, and the activation of diverse aging pathways, including loss of proteostasis, genomic instability, and ROS, in the endocrine system is cell type- and organ-specific. Thus, this raises the possibility that immune infiltration may also activate other aging pathways in addition to UPR.

In addition, the endocrine system plays a crucial role in maintaining overall homeostasis, and endocrine aging undoubtedly contributes to the aging of the entire organism. Endocrine organs may secrete age-related factors during the aging process, which could be capable of accelerating systemic aging. Therefore, future research will focus on identifying these aging-related factors secreted by the endocrine system and assessing their impact on individual aging.

## Supplementary data

Supplementary data is available at *Protein & Cell* online https://doi.org/10.1093/procel/pwaf074.

pwaf074_Supplementary_Figures_S1-S12

pwaf074_Supplementary_Tables_S1

pwaf074_Supplementary_Tables_S2

pwaf074_Supplementary_Tables_S3

## Data Availability

The raw sequence data of scRNA-seq and snRNA-seq were deposited at the Gene Expression Omnibus under accession code GSE239316.
