# Supplementary material for: A single-cell transcriptomic landscape characterizes the endocrine system aging in the mouse
Source: Protein Cell. 2025 Sep 1;17(1):27–45. doi: 10.1093/procel/pwaf074 (PMC12888919; doi:10.1093/procel/pwaf074)
Supplement: pwaf074_Supplementary_Figures_S1-S12 [file pwaf074_supplementary_figures_s1-s12.pdf]

Supplementary Materials

Materials and Methods

Experimental animals

SPF C57BL/6 mice were purchased from Beijing Vital River Laboratory Animal Technology Co., Ltd. and were fostered in the Laboratory Animal Center of Peking University in accordance with the regulations of the Laboratory Animal Management Committee of Peking University. SPF C57BL/6 mice were housed in a barrier environment with a constant temperature (20–25 °C), humidity (30–70%) and light exposure cycle (12 h light/dark per day). The data on the age of the two groups of C57BL6/J mice in our datasets of sex-shared endocrine organs are as follows: the young group (6-month-old, n = 12, 6 male and 6 female mice) and the aged group (24-month-old, n = 12, 6 male and 6 female mice). For the ovary, the young group (6-month-old, n = 6, 6 female mice) and the aged group (24-month-old, n = 6, 6 female mice) were used. For the testis, the young group (6-month-old, n = 6, 6 male mice) and the aged group (24-month-old, n = 6, 6 male mice) were analyzed.

Tissue dissociation and cell isolation

The mice were sacrificed, and the abdominal cavity of each mouse was opened. Positions above and below the entrance of the common duodenal bile duct were clamped with a pair of hemostatic clips, and 1–5 ml of cold 0.5 mg/ml collagenase solution was injected into the pancreas from the common bile duct via a syringe. Immediately after perfusion, the pancreases were removed from the abdominal cavities of the mice with a pair of forceps and placed into a 20 ml centrifuge tube containing 5 ml cold 0.5 mg/ml collagenase solution. Vials containing pancreatic organ were placed in a 37 °C water bath and incubated for 3 min. The centrifuge tube was gently shaken to gradually break the organ down until it was evenly dispersed. The digested product was filtered through a 0.25 mm nylon filter into a new 50 ml centrifuge tube, and the

filter was thoroughly cleaned with ice-cold PBS. The samples were subsequently centrifuged at 200 × g for 1 min, after which the supernatant was discarded. The organs were resuspended in cold PBS, and 5 ml of the organ suspension was poured into a 6 cm black-bottomed dish. Islets were removed with a 200 µL pipette and transferred to a 1.5 mL tube containing a small amount of cold PBS. Tubes containing pancreatic organ or islets were centrifuged at 200 × g for 1 min at 4 °C, and the supernatant was discarded. The pellets were resuspended in 0.25% trypsin EDTA solution and incubated in a 37 °C water bath. After a 4 min incubation, the pellets were dissociated by gentle aspiration with a 200 µl pipette tip for 1 min. Digestion was stopped by adding 0.4 volumes of cold fetal bovine serum (FBS) and gently mixing the sample. The plates were subsequently centrifuged at 250 × g for 3 min at 4 °C. The supernatant was discarded. The cells were subsequently resuspended in Hanks buffer (pH 7.4) containing 1% FBS.

The hypothalamus, pituitary gland, pineal gland, thyroid gland and adrenal gland were subsequently removed from the brain, thoracic cavity or abdominal cavity of each mouse. The pituitary and pineal glands were cut into small pieces using surgical scissors and incubated with collagenase (0.5% in HBSS) at 37 °C for 5–15 min (depending on the type of organ) until they were dissociated into a single-cell suspension. The digested product was filtered through a 40 µm nylon filter into a new 50 ml centrifuge tube and washed with cold PBS. The cells were subsequently resuspended in Hanks buffer (pH 7.4) containing 1% FBS.

Adrenal glands were treated with 1 mg/mL collagenase (in PBS) and sheared using a 1-mL pipette on ice until a single-cell suspension was generated. The digested product was filtered through a 40 µm nylon filter into a new 50 ml centrifuge tube and washed with cold PBS. The cells were subsequently resuspended in Hanks buffer (pH 7.4) containing 1% FBS.

The thyroid glands were cut into small pieces using surgical scissors and incubated with type II collagenase (0.5% in HBSS) at 37 °C for 5–15 min until they were dissociated into a single-cell suspension. The digested product was filtered through a 40 μm nylon filter into a new 50 ml centrifuge tube and washed with cold PBS. The cells were subsequently resuspended in Hanks buffer (pH 7.4) containing 1% FBS.

For the testes, the tunica albuginea was removed and seminiferous tubules were transferred to 10ml digestion buffer 1 (DMEM: F12 medium, 200 μg/ml collagenase IA and 400 units /ml DNase I). The seminiferous tubules were gently shaken by hand to disperse and then allowed to stand for 1 min at room temperature. The seminiferous tubules were then transferred to digestion buffer 2 (DMEM:F12 media, 200 μg/ml trypsin and 400 units/ml DNaseI) and dissociated for 5 min at 35℃, after which the digestion was terminated using fetal calf serum(FBS). Cells were filtered through a 40 μm filter, washed in phosphate buffer (PBS), centrifuged at 600g for 3min, and cells were resuspended in Hanks buffer (pH 7.4) containing 1% FBS.

Ovaries were mechanically dissected and treated with 1 mg/mL collagenase type IV(0.5% in HBSS) for 20 min at 37 ° C. The digested product was filtered through a 40 μm nylon filter into a new 50 ml centrifuge tube, and wished with cold PBS. Then cells were resuspended in Hanks buffer (pH 7.4) containing 1% FBS.

Tissue embedding and cryosectioning

The mice were anesthetized and perfused with 20 ml PBS and 20 ml 4% paraformaldehyde through the left ventricle. After perfusion, the required endocrine organs were separated and fixed in 4% paraformaldehyde at 4 °C overnight in a organ block for a 4% paraformaldehyde volume ratio of 1:7. After fixation, the blocks were successively placed in 10% sucrose solution, 20% sucrose solution or 30% sucrose solution for dehydration at 4 °C. The dehydrated organ was subsequently placed in an embedding box and immersed in OCT compound. The embedding box was flash-frozen

in a freezing microtome. Frozen organ blocks were sectioned to a thickness of 10 μm using a freezing microtome. The organ sections were transferred to slides and stored at -20 °C until use.

Immunofluorescence

The organ samples were allowed to warm for 30 min at room temperature. The samples were subsequently fixed with 4% paraformaldehyde for 15 min at room temperature, washed three times for 5 min each with PBS at room temperature, and treated with 0.5% Triton X-100 on ice for 15 min. The samples were subsequently washed three times with PBS for 5 min each at room temperature. After the last wash, the slides were transferred to a humid chamber. The samples were blocked with 1% BSA for 30 min at room temperature, washed three times with PBS for 5 min each, and then incubated with 100–150 μl primary antibody diluted in PBST (PBS + 0.1% Tween 20) overnight at 4 °C. Then, the samples were washed 4 times with PBST (1 PBS + 0.1% Triton X-100) for 5 min each and incubated with 100–150 μl secondary antibody diluted in PBST (PBS + 0.1% Tween 20) for 1.5 h at room temperature in the dark. The samples were washed three times with PBST (1 PBS + 0.1% Triton X-100) for 5 min each time and then washed once with 1x PBS. Then, diluted DAPI (final concentration of 1–2 ng/μl) was added, and the samples were incubated for 3–5 min at room temperature. The samples were washed three times with 1 ml PBS for 5 min each and once with 1 ml ddH2O. Finally, the slides were mounted with 10 μl Fluoromount-G and observed under a fluorescence microscope after 1 h.

Western blotting

Each protein sample was added to SDS loading buffer and incubated in a metal bath at 95 °C for 5 min for denaturation. A 15% resolving gel and spacer gel were prepared and successively added to a mold to prepare for SDS–PAGE. Electrophoresis was performed at 82 V for 30 min and then increased to 135 V for 90 min. The

polyacrylamide gel, filter paper, and cellulose acetate membranes were immersed in transfer buffer for 5 min, placed in a transfer chamber and transferred at a constant voltage of 19 V for 2 h. The cellulose acetate membrane was washed 3 times with TBST for 5 min each, blocked in blocking solution for 1 h, and incubated with primary antibody at 4 °C overnight. After incubation with the primary antibody, the membrane was washed with TBST 3 times for 5 min each. The membrane was incubated with secondary antibody at 37 °C for 1 h and then washed with TBST 3 times for 5 min each. Finally, an Odyssey® M Imaging System was used to obtain images.

Isolation and culture of pituitary cells

After isolation of the pituitary glands from 6-month-old male C57BL/6J mice, the anterior pituitary was dissociated into single cells by continuous incubation with collagenase (0.5% in HBSS). A total of 60,000 cells were isolated from each pituitary gland. Freshly isolated cells were seeded at a density of 75,000–100,000 cells/ml in 6 cm dishes of DMEM/F12 containing 0.5% BSA, B27 and N2 and 20 ng/ml bFGF and EGF. The growth factor solution was replenished daily, and the medium was replaced after 3 days.

Knockdown and overexpression of H2-Kb in vitro

To investigate the role of GZMK and H2-Kb in the aging process of the endocrine system, we isolated and cultured mouse pituitary cells in vitro. H2-Kb expression was subsequently knocked down via shRNA or overexpressed using the TetOn system to explore the function of H2-Kb. The TetOn system is an inducible system that can be transfected into cells to overexpress target proteins in the presence of doxycycline (DOX). Then, mouse pituitary cells cultured in vitro were treated with 100 ng/ml GZMK to explore whether the expression of ATF4, H2-Kb and p16 was significantly upregulated. Knockdown of H2-Kb expression via shRNA was used to explore whether

H2-Kb could partially block the upregulation of ATF4 and p16 expression caused by GZMK treatment.

GZMK intervention in mice in vivo

To further examine whether GZMK also plays a key role in systemic aging in mice, we injected GZMK and molecular inhibitors of GZMK downstream receptors into mice. To inhibit GZMK signaling, we used the small molecule inhibitors resatorvid and SCH79797 to inhibit activity of the GZMK receptors TLR4 and PAR1, respectively. The mice were divided into five groups—aged control, aged GZMK injection, aged anti-GZMK, young control, and young GZMK injection—with three biological replicates in each group. We used 6-month-old male mice as the young group and 24-month-old male mice as the old group. Young and aged mice in the GZMK-injected group were injected with 300 μg/kg GZMK per injection every 3 days for a total of 4 weeks. In the anti-GZMK group, 3 mg/kg resatorvid and 30 μg/kg SCH79797 were injected every 3 days for a total of 4 weeks. In the control group, young and aged mice were injected with equal volumes of saline once every 3 days for 4 weeks. The expression levels of p16, H2-Kb, XBP1 and ATF4 in the pituitary, thyroid, and adrenal glands were measured by immunofluorescence after GZMK intervention in mice.

Enzyme-linked immunosorbent assay

For the serum ELISA assay, we used a commercially available double-antibody sandwich ELISA kit. Blood was collected from mice, and serum was separated by centrifugation at 3000 rpm for 10 minutes. The ELISA was performed according to the manufacturer’ s instructions. Briefly, the ELISA plate was coated with the capture antibody overnight at 4°C, followed by blocking with 1% BSA in PBS for 1 hour. Serum samples, along with standards, were added to the wells and incubated for 1-2 hours at room temperature. After washing, a detection antibody conjugated to an enzyme was added, followed by further incubation. The substrate solution was added,

and the color development was measured at 450 nm using a microplate reader. The concentrations of the target analytes in the serum were determined using a standard curve.

Rotarod Test

The rotarod test was used to assess motor coordination and balance. Mice were placed on an accelerating rotarod which increased from 4 to 40 rpm over 10 minutes. The test was performed for three trials with a 30-minute interval between each trial. Latency to fall (the time spent on the rod before falling) was recorded for each trial. The average latency for each mouse was used for analysis.

Y-Maze Test

The Y-maze test was used to assess spatial working memory and exploratory behavior. The Y-maze apparatus consisted of three arms (30 cm length × 5 cm width × 15 cm height), arranged at a 120° angle from each other. Mice were placed at the end of one arm and allowed to explore freely for 10 minutes. The total number of arm entries and the number of spontaneous alternations (i.e., the mouse visiting three different arms consecutively without returning to the previous one) were recorded.

Open-Field Test

The open-field test was used to assess general locomotor activity and anxiety-like behavior. The open-field apparatus was a square arena (40 cm × 40 cm) with low walls (25 cm). Mice were placed in the center of the arena and allowed to explore freely for 10 minutes. Total distance traveled and time spent in the center versus the periphery were recorded using a video tracking system. Time spent in the center of the arena was used as an indicator of anxiety-like behavior, with decreased central exploration indicating increased anxiety.

Quality control, data processing and analysis

We employed CellRanger (version 6.1.2) to align the sequence obtained from 10x Genomics to the mm10 (2020) reference genome. For the hypothalamic nuclei data, we utilized the "--include-introns" parameter, whereas default parameters were used for the single-cell data from other organs. The resulting feature–barcode matrices were read into R v.4.1.2, and we used Seurat (v.4.1.1)(Stuart et al. 2019) to filter low-quality cells. Cells with fewer than 500 detected genes and fewer than 500 unique molecular identifiers were excluded. For all the organs, we removed the cells or nuclei whose ribosomal proportions were greater than 40%. Cells or nuclei with a high percentage of mitochondrial genes (>10%) were removed (the threshold for cells of the testis was a mitochondrial gene ratio of 20%) All QC metrics for all organs are summarized in Table S1. We then processed each sample via the tutorial of the DoubletFinder (version 2.0.3)(McGinnis et al. 2019) package to detect and filter doublets.

Clustering and cell type identification

We performed dimensionality reduction, clustering, and annotating of the data from six endocrine organs separately via Seurat. Each filtered dataset was normalized by a scale factor (10,000) via the Seurat “NormalizeData” function. Next, we used the "FindVariableFeatures" function to identify the most variably expressed genes. We then applied the "ScaleData" function to normalize the expression values of these features, ensuring that each gene contributed equally to downstream analyses and removing any potential biases introduced during library preparation and sequencing. Principal component analysis (PCA) was then applied via “RunPCA” in Seurat to identify the most significant sources of variation in the data. To remove the batch effects between different samples within the dataset, harmony dimension reduction with the RunHarmony function in Harmony (v.0.1.0)(Korsunsky et al. 2019) was performed via PCA. The first 30 dimensions of Harmony were selected for subsequent analysis.

Dimensionality reduction and clustering were performed via the "RunUMAP," "FindNeighbors," and "FindClusters" functions in Seurat to generate a low-dimensional representation of the cells and identify distinct cell populations. For differential expression analysis within each cluster, we utilized the "FindAllMarkers" function from Seurat to identify genes that were differentially expressed in specific cell clusters compared with all other cells in the dataset. We excluded low-quality cell clusters with high expression of *Gm42418* or *AY036118.* The cell types were annotated via marker genes; the marker genes for each cell type are provided in Table S2. To gain a more detailed classification of immune cells, we reclustered the clusters of Ptprc+ cells to establish an immune cell atlas for each organ (except the hypothalamus).

Integration of publicly available datasets

To assess the quality of our data and evaluate the accuracy of the annotations for the major cell types, we integrated public hypothalamus, pituitary, adrenal, and pancreatic islet datasets(Ruf-Zamojski et al. 2021; Hajdarovic et al. 2022; Hrovatin et al. 2023; Li et al. 2024). We downloaded hypothalamus, pituitary and pancreatic islet matrices from the following Gene Expression Omnibus datasets: GSE188646, GSE151962, and GSE211799. The adrenal gland expression dataset used for scRNA-seq was downloaded from OMIX at OMIX001083. Before integration, we excluded data from mice with diseases such as diabetes and retained only data from healthy adult mice to exclude the effects of disease and developmental effects. Public datasets from different endocrine organs were integrated with our data via the “FindIntegrationAnchors” function and the “IntegrateData” function of the Seurat package. Dimensionality reduction and clustering were performed via the “RunUMAP,” “FindNeighbors,” and “FindClusters” functions and via visualization to assess the consistency of the public data and our data cell type mapping. Cell markers from different dataset sources for the same cell type were calculated via the “FindAllMarkers” function, and the similarity was assessed via the hypergeometric test as previously described(Kuppe et al. 2022). To validate the findings in the ovary and testis, we reanalyzed publicly available

ovarian(Winkler et al. 2024) and testicular(Zhang et al. 2023) datasets (E-MTAB-11491, E-MTAB-12889 and OMIX1000) to confirm the consistency of our conclusions.

Differential expression and cell type-specific aging-associated DEG analyses

Differential expression analysis for each cell type between different groups (young group vs. aged group) was conducted via the Wilcoxon rank-sum test as implemented in the "FindMarkers" function of the Seurat package (version 4.1.1). All cell types considered in the differential gene expression analysis were present in both the young and aged groups. We defined genes with logFC > 0.25 and adjusted p value < 0.05 as aging- associated upregulated differentially expressed genes. Conversely, genes with logFC < -0.25 and adjusted p value <0.05 were defined as aging-associated downregulated differentially expressed genes. The combination of genes with upregulated and downregulated expression constitutes the set of differentially expressed genes associated with aging (aging-associated DEGs).

Gene set score analysis

To assess the immune response in various endocrine organs, we employed the “AddModuleScore” function in Seurat to score the cells on the basis of the SASP-related gene set from SenMayo(Liberzon et al. 2015).

Assessing the impact of aging pathways

To assess the impact of aging on different types of functional endocrine cells, we initially performed GO analysis on the aging-associated DEGs in these cells and categorized the GO terms according to aging pathways. We scored the functional endocrine cells using the corresponding aging pathway-related gene sets with the AddModuleScore function in Seurat, assigning each cell a score indicating its activity in the respective aging pathway. To measure the differences between young and aged

groups within each endocrine cell type, we applied Cohen's d to quantify the effect size of the difference between the two groups (YM/OM or YF/OF) 58. A larger Cohen's d value indicates a greater difference between the young and aged groups, highlighting the extent of the age-related changes within each endocrine cell type. First, we separately calculated the means ($M_{y,p,c}$ for the young group, $M_{o,p,c}$ for the aged group) and standard deviations ($S_{y,p,c}$ for the young group, $S_{o,p,c}$ for the aged group) of the scores of the aging pathways p for each endocrine cell type c in females or males. We defined the effective size $d_{p,c}$ for aging pathway p in cell type c (consisting of $N_{y,p,c}$ cells from young individuals and o $N_{o,p,c}$ cells from old individuals) as

$$\frac{M_{o,p,c} - M_{y,p,c}}{S_{pooled}}$$

, where

$$S_{pooled} = \sqrt{\frac{(N_{o,p,c} - 1)S^2_{o,p,c} + (N_{y,p,c} - 1)S^2_{y,p,c}}{N_{o,p,c} + N_{y,p,c} - 2}}$$

.

Single-cell regulatory network inference and clustering (SCENIC)

SCENIC is a tool used to uncover gene regulatory networks by predicting transcription factor activities and target genes(Aibar et al. 2017). To optimize computational efficiency, we utilized the pySCENIC(Van de Sande et al. 2020) implementation of the SCENIC pipeline to infer the core transcription factors and regulatory networks of aging-associated DEGs separately in male and female thyroid follicular cells. First, we inferred the coexpression matrix of aging-associated DEGs in thyroid follicular cells using the GRNBoost algorithm. Subsequently, we pruned the indirect targets of these gene modules using cisTarget with mm10-based gene databases. Finally, we quantified the activity of these regulons using AUCell. Transcription factors and their target aging-associated DEGs were visualized using Cytoscape (3.9.1)(Shannon et al. 2003).

GO analysis

We utilized the ClusterProfiler (version 4.2.2) package in R to perform Gene Ontology (GO) enrichment analysis. Significantly enriched GO terms were identified on the basis of a p value threshold of less than 0.05(Moskalev et al. 2012).

Cell–cell communication analysis

CellChat (version 1.6.1) was employed to detect major alterations in the interactions between immune cells and endocrine cells during the aging process(Jin et al. 2021). We initially ran CellChat separately on the young and aged cells from each organ and subsequently merged the different CellChat objects together. We conducted a differential expression analysis between two biological conditions (aged and young) for each cell group and then identified upregulated and downregulated signaling pathways on the basis of the probabilities of ligand–receptor interactions. All p value thresholds were set at 0.05 or lower.

Prediction of functional endocrine cell age using machine learning

To mitigate the influence of different gene threshold settings and identify markers of aging in endocrine cells, we employed machine learning algorithms and interpretability methods for functional endocrine cells. XGBoost (eXtreme Gradient BOOSTing) is a machine learning algorithm that is widely used for classification task(Chen et al. 2016). When analyzing aging-related characteristics of endocrine cells, XGBoost effectively captured nonlinear relationships and interactions between features while offering high predictive accuracy (Figure 7C). Additionally, the built-in feature importance evaluation in XGBoost helped us identify key features that had the most significant impact on the aging process.The interpretability of the model allowed us to better understand functional changes in endocrine cells. Functional endocrine cells were classified into young and aging groups using the XGBoost algorithm. The datasets were divided into a training set (80%) and a testing set (20%) to train and evaluate the classification models. To elucidate the importance of features in the model, we

employed the gain of XGBoost and SHAP methods(Lundberg and Lee 2017). The "gain" metric in XGBoost is utilized to measure the priority of feature splitting, indicating the extent to which model performance improves when selecting a specific feature for partitioning. SHAP values provide insights into the impact of each feature on predictions related to both youthful and aging states. We took the intersection of the top 20 features by SHAP values and the top 20 features by gain as the important features, and used normalized gain as the indicator of importance. The pipeline and functions were implemented in Python using the Scikit-learn library and the SHAP package.

Statistical analysis

GraphPad Prism (version 8.0.2) (GraphPad Software, CA, USA) was used for statistical analyses of immunofluorescence. All bar graphs indicate the mean ± the standard error of the mean (SEM). Two-tailed unpaired Student's t-test was used to compares two groups of data. The biological replicate number of animals and cells in each group are indicated in the figure legends, with P values for comparisons shown in the figures. All experiments were repeated at least three times with similar results. Statistical analyzes of sc/snRNA-seq were conducted using R. The hypergeometric test and Wilcoxon test were used to determine statistical significance.

References

Aibar, S., González-Blas, C. B., Moerman, T., Huynh-Thu, V. A., Imrichova, H., Hulselmans, G., Rambow, F., Marine, J. C., Geurts, P., Aerts, J., van den Oord, J., Atak, Z. K., Wouters, J. and Aerts, S. (2017) SCENIC: single-cell regulatory network inference and clustering. *Nat Methods* 14 (11), 1083-1086.

Chen, T., Guestrin, C. J. P. o. t. n. A. S. I. C. o. K. D. and Mining, D. (2016) XGBoost: A Scalable Tree Boosting System.

Hajdarovic, K. H., Yu, D., Hassell, L.-A., Evans, S. A., Packer, S., Neretti, N. and Webb, A. E. (2022) Single-cell analysis of the aging female mouse hypothalamus. *Nature Aging* 2 (7), 662-678.

Hrovatin, K., Bastidas-Ponce, A., Bakhti, M., Zappia, L., Büttner, M., Salinno, C., Sterr, M., Böttcher, A., Migliorini, A., Lickert, H. and Theis, F. J. (2023) Delineating mouse β-cell identity during lifetime and in diabetes with a single cell atlas. *Nat Metab* 5 (9), 1615-1637.

Jin, S., Guerrero-Juarez, C. F., Zhang, L., Chang, I., Ramos, R., Kuan, C.-H., Myung, P., Plikus, M. V. and Nie, Q. (2021) Inference and analysis of cell-cell communication using CellChat. *Nature Communications* 12 (1).

Korsunsky, I., Millard, N., Fan, J., Slowikowski, K., Zhang, F., Wei, K., Baglaenko, Y., Brenner, M., Loh, P.-r. and Raychaudhuri, S. (2019) Fast, sensitive and accurate integration of single-cell data with Harmony. *Nature Methods* 16 (12), 1289-1296.

Kuppe, C., Ramirez Flores, R. O., Li, Z., Hayat, S., Levinson, R. T., Liao, X., Hannani, M. T., Tanevski, J., Wünnemann, F., Nagai, J. S., Halder, M., Schumacher, D., Menzel, S., Schäfer, G., Hoeft, K., Cheng, M., Ziegler, S., Zhang, X., Peisker, F., Kaesler, N., Saritas, T., Xu, Y., Kassner, A., Gummert, J., Morshuis, M., Amrute, J., Veltrop, R. J. A., Boor, P., Klingel, K., Van Laake, L. W., Vink, A., Hoogenboezem, R. M., Bindels, E. M. J., Schurgers, L., Sattler, S., Schapiro, D., Schneider, R. K., Lavine, K., Milting, H., Costa, I. G., Saez-Rodriguez, J. and Kramann, R. (2022) Spatial multi-omic map of human myocardial infarction. *Nature* 608 (7924), 766-777.

Li, F., Xing, X., Jin, Q., Wang, X.-M., Dai, P., Han, M., Shi, H., Zhang, Z., Shao, X., Peng, Y., Zhu, Y., Xu, J., Li, D., Chen, Y., Wu, W., Wang, Q., Yu, C., Chen, L., Bai, F. and Gao, D. (2024) Sex differences orchestrated by androgens at single-cell resolution. *Nature* 629 (8010), 193-200.

Liberzon, A., Birger, C., Thorvaldsdóttir, H., Ghandi, M., Mesirov, J. P. and Tamayo, P. (2015) The Molecular Signatures Database Hallmark Gene Set Collection. *Cell Systems* 1 (6), 417-425.

Lundberg, S. M. and Lee, S.-I. (2017) A unified approach to interpreting model predictions. In *Proceedings of the 31st International Conference on Neural Information Processing Systems.* Long Beach, California, USA. Curran Associates Inc. 4768–4777.

McGinnis, C. S., Murrow, L. M. and Gartner, Z. J. (2019) DoubletFinder: Doublet Detection in Single-Cell RNA Sequencing Data Using Artificial Nearest Neighbors. *Cell Systems* 8 (4), 329-337.e4.

Moskalev, A. A., Smit-McBride, Z., Shaposhnikov, M. V., Plyusnina, E. N., Zhavoronkov, A., Budovsky, A., Tacutu, R. and Fraifeld, V. E. (2012) Gadd45 proteins: relevance to aging, longevity and age-related pathologies. *Ageing Res Rev* 11 (1), 51-66.

Ruf-Zamojski, F., Zhang, Z., Zamojski, M., Smith, G. R., Mendelev, N., Liu, H., Nudelman, G., Moriwaki, M., Pincas, H., Castanon, R. G., Nair, V. D., Seenarine, N., Amper, M. A. S., Zhou, X., Ongaro, L., Toufaily, C., Schang, G., Nery, J. R., Bartlett, A., Aldridge, A., Jain, N., Childs, G. V., Troyanskaya, O. G., Ecker, J. R., Turgeon, J. L., Welt, C. K., Bernard, D. J. and Sealfon, S. C. (2021) Single nucleus multi-omics regulatory landscape of the murine pituitary. *Nature Communications* 12 (1), 2677.

Shannon, P., Markiel, A., Ozier, O., Baliga, N. S., Wang, J. T., Ramage, D., Amin, N., Schwikowski, B. and Ideker, T. (2003) Cytoscape: a software environment for integrated models of biomolecular interaction networks. *Genome Res* 13 (11), 2498-504.

Stuart, T., Butler, A., Hoffman, P., Hafemeister, C., Papalexi, E., Mauck, W. M., Hao, Y., Stoeckius, M., Smibert, P. and Satija, R. (2019) Comprehensive Integration of Single-Cell Data. *Cell* 177 (7), 1888-1902.e21.

Van de Sande, B., Flerin, C., Davie, K., De Waegeneer, M., Hulselmans, G., Aibar, S., Seurinck, R., Saelens, W., Cannoodt, R., Rouchon, Q., Verbeiren, T., De Maeyer, D., Reumers, J., Saeys,

Y. and Aerts, S. (2020) A scalable SCENIC workflow for single-cell gene regulatory network analysis. *Nat Protoc* 15 (7), 2247-2276.
Winkler, I., Tolkachov, A., Lammers, F., Lacour, P., Daugelaite, K., Schneider, N., Koch, M.-L., Panten, J., Grünschläger, F., Poth, T., Ávila, B. M. d., Schneider, A., Haas, S., Odom, D. T. and Gonçalves, Â. (2024) The cycling and aging mouse female reproductive tract at single-cell resolution. *Cell* 187 (4), 981-998.e25.
Zhang, W., Xia, S., Xiao, W., Song, Y., Tang, L., Cao, M., Yang, J., Wang, S., Li, Z., Xu, C., Liu, J., Zhao, S., Yang, C. and Wang, J. (2023) A single-cell transcriptomic landscape of mouse testicular aging. *Journal of Advanced Research* 53, 219-234.

Supplementary Figures and Supplementary Figure legends

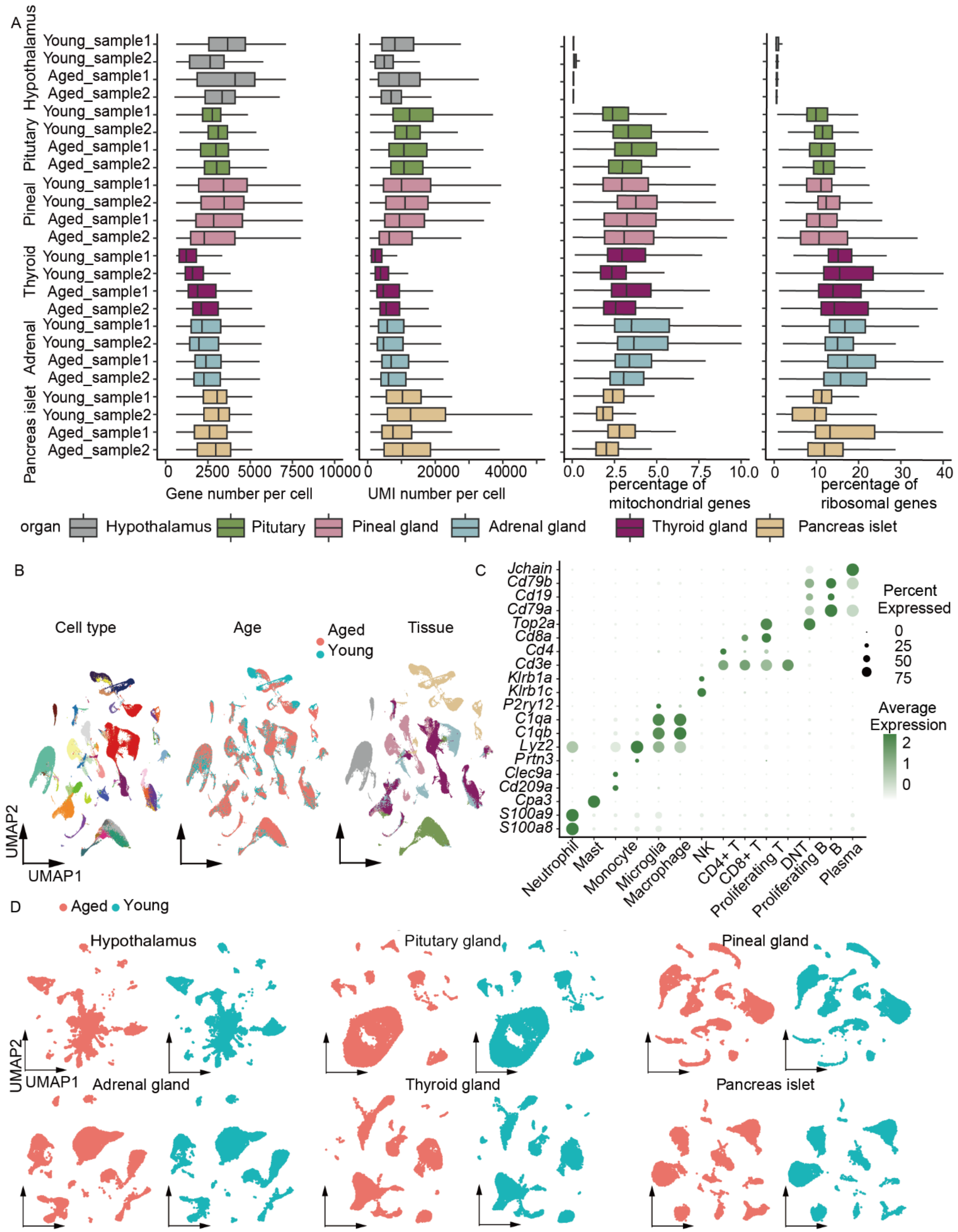

Figure S1 Construction of a single-cell transcriptomic atlas of multiorgan endocrine aging (related to Figure 1). (A) Box plots showing the gene number, unique molecular identifier (UMI) number per cell, percentage of mitochondrial genes and percentage of ribosomal genes in the indicated organs across the young and aged groups. **(B)** UMAP plots showing cell types that integrate the six endocrine organs (left), distribution of cells from the young and aged groups (middle) and distribution of cells from different endocrine organs (right) marked in different colors. The color coding of different cell types is consistent with that in Figure 1B, and that of organ types is in accordance with that in Figure S1A. **(C)** Dot plot showing the marker gene expression levels of immune cell subpopulations within the indicated organs. **(D)** UMAP plots of the young and old groups for each tissue, corresponding to Figure 1B.

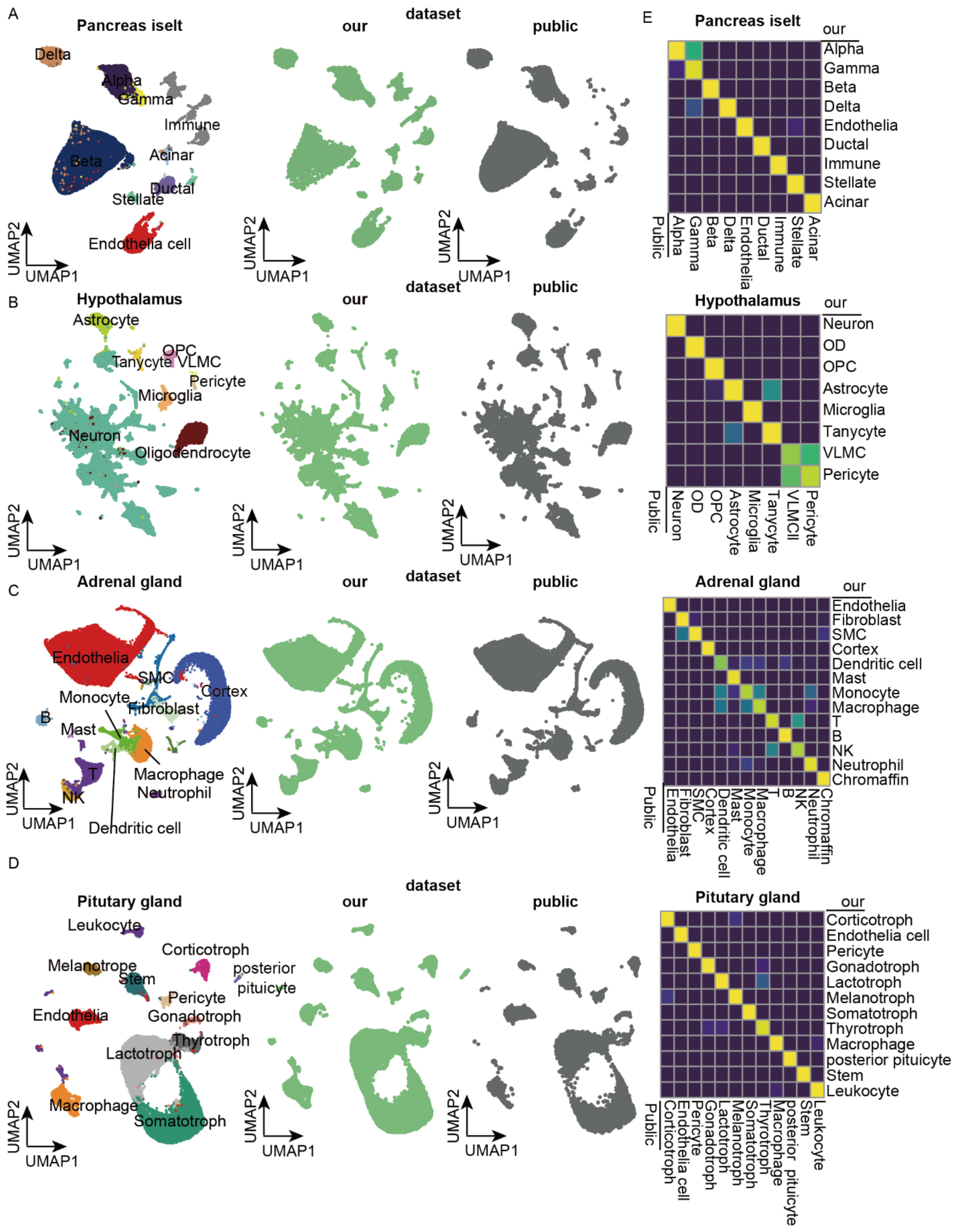
A
Pancreas iselt
dataset
our
public
Delta
Alpha
Gamma
Immune
Beta
Acinar
Ductal
Stellate
Endothelia cell
UMAP2
UMAP1
E
Pancreas iselt
our
Alpha
Gamma
Beta
Delta
Endothelia
Ductal
Immune
Stellate
Acinar
Public
B
Hypothalamus
Astrocyte
OPC
Tanycyte
VLMC
Pericyte
Microglia
Neuron
Oligodendrocyte
Hypothalamus
Neuron
OD
OPC
Astrocyte
Microglia
Tanycyte
VLMC
Pericyte
VLMCII
C
Adrenal gland
Endothelia
SMC
Cortex
Monocyte
Fibroblast
B
Mast
T
NK
Macrophage
Neutrophil
Dendritic cell
Endothelia
Fibroblast
SMC
Cortex
Dendritic cell
Mast
Monocyte
Macrophage
T
B
NK
Neutrophil
Chromaffin
D
Pituitary gland
Leukocyte
Corticotroph
Melanotrope
Stem
posterior
pituicyte
Pericyte
Endothelia
Gonadotroph
Thyrotroph
Lactotroph
Macrophage
Somatotroph
Corticotroph
Endothelia cell
Pericyte
Gonadotroph
Lactotroph
Melanotroph
Somatotroph
Thyrotroph
Macrophage
posterior pituicyte
Stem
Leukocyte

Figure S2 Integration and comparison of the aging endocrine organ atlas with public datasets (related to Figure 1).

(A-D) UMAP plots demonstrating the integration of public data with our data (left) and the distribution of cells from the public dataset and our data (middle and right) for the pancreatic islets **(A)**, hypothalamus **(B)**, adrenal glands **(C)** and pituitary glands **(D)**. **(E)** Heatmaps demonstrating the similarity of our data cell types to public data in the indicated endocrine organs. The similarity was calculated as the negative logarithm of the adjusted p value for the overlap of the top gene markers for each cell type between the different datasets (hypergeometric test).

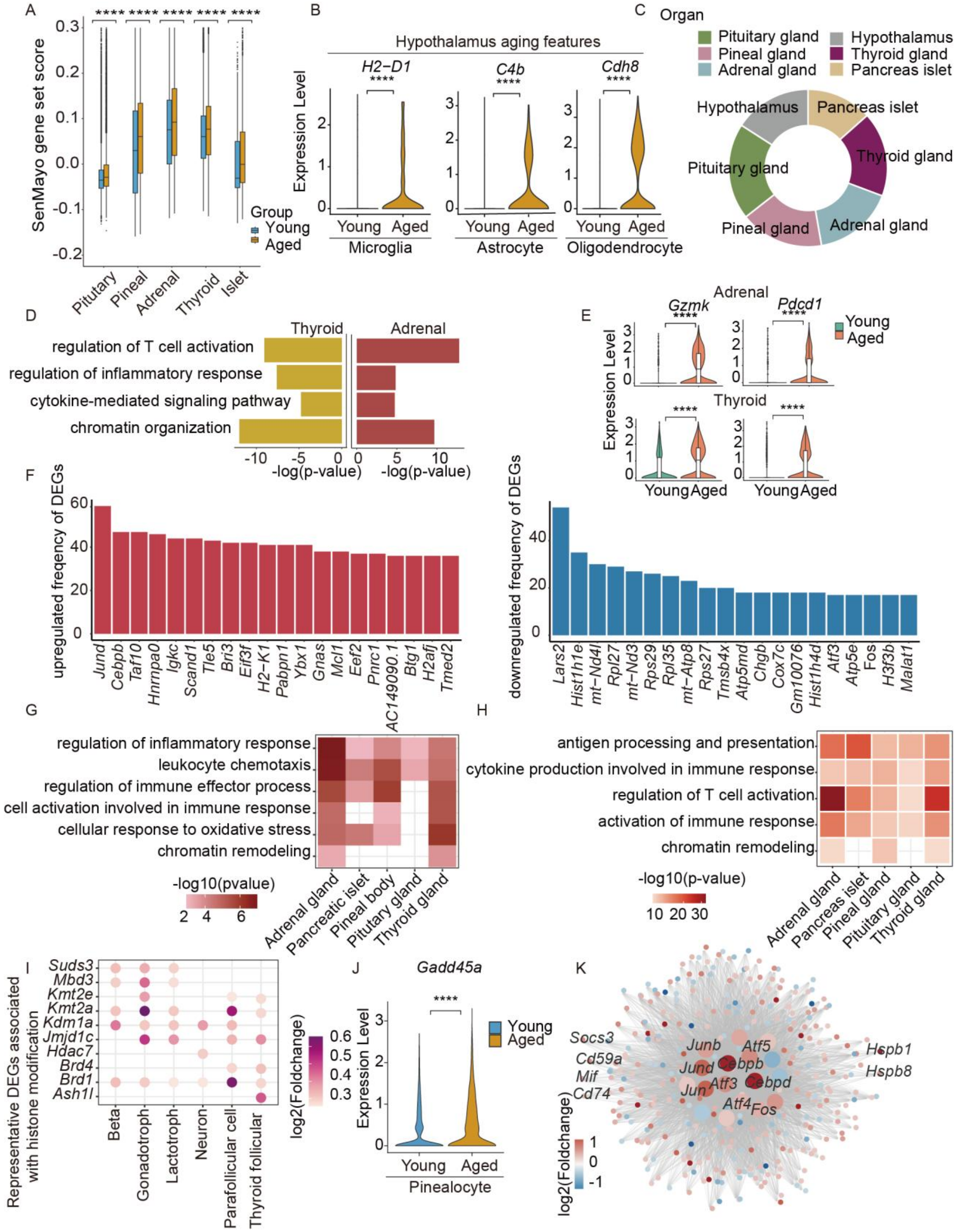
A
SenMayo gene set score
****
Pituitary
Pineal
Adrenal
Thyroid
Islet
Group
Young
Aged
B
Hypothalamus aging features
H2−D1
C4b
Cdh8
Expression Level
Young
Aged
Microglia
Astrocyte
Oligodendrocyte
C
Organ
Pituitary gland
Pineal gland
Adrenal gland
Hypothalamus
Thyroid gland
Pancreas islet
D
Thyroid
Adrenal
regulation of T cell activation
regulation of inflammatory response
cytokine-mediated signaling pathway
chromatin organization
-log(p-value)
E
Adrenal
Thyroid
Gzmk
Pdcd1
Expression Level
Young
Aged
F
upregulated freqency of DEGs
Jund
Cebpb
Taf10
Hnrnpa0
Igkc
Scand1
Tle5
Bri3
Eif3f
H2−K1
Pabpn1
Ybx1
Gnas
Mcl1
Eef2
Pnrc1
AC149090.1
Btg1
H2afj
Tmed2
downregulated frequency of DEGs
Lars2
Hist1h1e
mt−Nd4l
Rpl27
mt−Nd3
Rps29
Rpl35
mt−Atp8
Rps27
Tmsb4x
Atp5md
Chgb
Cox7c
Gm10076
Hist1h4d
Atf3
Atp5e
Fos
H3f3b
Malat1
G
regulation of inflammatory response
leukocyte chemotaxis
regulation of immune effector process
cell activation involved in immune response
cellular response to oxidative stress
chromatin remodeling
-log10(pvalue)
Adrenal gland
Pancreatic islet
Pineal body
Pituitary gland
Thyroid gland
H
antigen processing and presentation
cytokine production involved in immune response
regulation of T cell activation
activation of immune response
chromatin remodeling
-log10(p-value)
Adrenal gland
Pancreas islet
Pineal gland
Pituitary gland
Thyroid gland
I
Representative DEGs associated with histone modification
Suds3
Mbd3
Kmt2e
Kmt2a
Kdm1a
Jmjd1c
Hdac7
Brd4
Brd1
Ash1l
Beta
Gonadotroph
Lactotroph
Neuron
Parafollicular cell
Thyroid follicular
log2(Foldchange)
J
Gadd45a
Expression Level
Young
Aged
Pinealocyte
K
Socs3
Cd59a
Mif
Cd74
Junb
Atf5
Jund
Cebpb
Atf3
Cebpd
Jun
Atf4
Fos
Hspb1
Hspb8
log2(Foldchange)

Figure S3 Aging reshaped the transcriptomic landscape of diverse endocrine organs (related to Figure 2 and Figure 3). (A) Box plots representing the SenMayo gene set scores for the young and aged groups of the indicated organs. **(B)** Violin diagram showing expression changes in previously reported hypothalamic aging-related genes in the indicated cell types. **(C)** The donut chart illustrating the weights of cell types from different organs. **(D)** Bar plots showing aging pathways in the adrenal gland and thyroid gland. **(E)** Violin plots showing that *Gzmk* and *Pdcd1* were upregulated during aging. **(F)** Bar plots showing the frequencies of the top 20 upregulated (left) and downregulated (right) aging-associated DEGs observed across all cell types in the six organs. **(G-H)** Heatmaps showing the aging pathways of endothelial cells and immune cells across different endocrine organs. **(I)** The dot plot showing representative DEGs related to histone modifications in the indicated functional endocrine cells. **(J)** The violin plot demonstrating expression levels of the oxidative stress sensor gene *Gadd45a* in pinealocytes from young and aged groups. **(K)** The network visualization of transcription factor regulation of aging-associated DEGs in thyrotrophs.

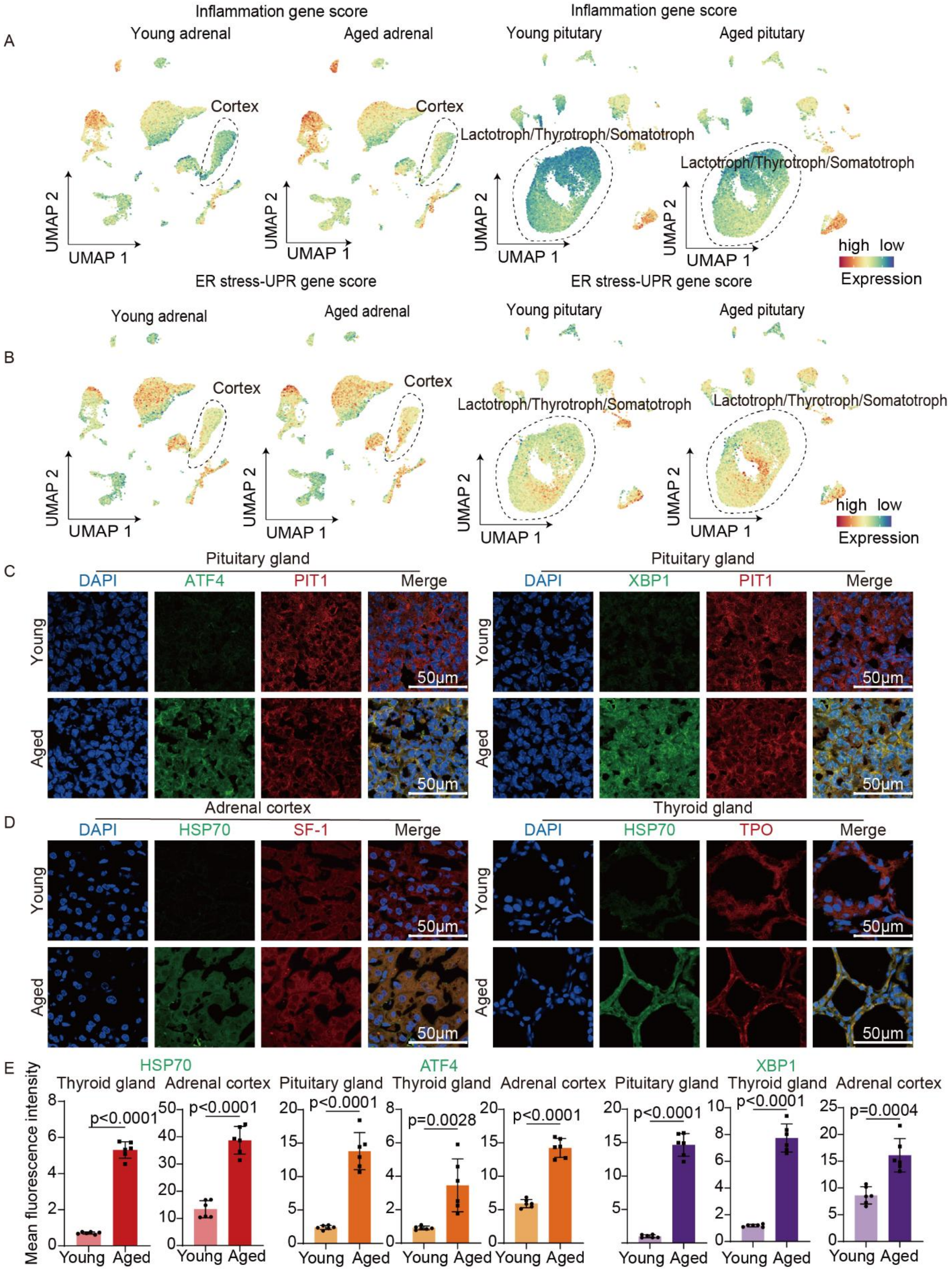
A
Inflammation gene score
Young adrenal
Aged adrenal
Cortex
Young pituitary
Aged pituitary
Lactotroph/Thyrotroph/Somatotroph
UMAP 1
UMAP 2
high low
Expression
B
ER stress-UPR gene score
C
Pituitary gland
DAPI
ATF4
PIT1
XBP1
Merge
Young
Aged
50μm
D
Adrenal cortex
Thyroid gland
HSP70
SF-1
TPO
E
Mean fluorescence intensity
p<0.0001
p=0.0028
p=0.0004
Adrenal cortex
Pituitary gland
Thyroid gland

Figure S4 The UPR and inflammation were enhanced in multiple functional endocrine cells (related to Figure 3). (A-B) UMAP plots showing ER stress associated inflammation **(A)** and UPR scores **(B)** across young and aged in the adrenal glands and pitutary glands. **(C)** Representative immunofluorescence images of ATF4 or XBP1 coexpression with PIT across the indicated organs in the young and aged groups (n = 6). PIT1 expression can distinguish pituitary thyrotrophs, somatotrophs and lactotrophs from other cell types. **(D)** Representative immunofluorescence images of HSP70 coexpression with SF-1 or TPO across the indicated organs in the young and aged groups (n = 6). **(E)** Quantification of HSP70, ATF4 and XBP1 across the indicated organs in the young and aged groups (n = 6).

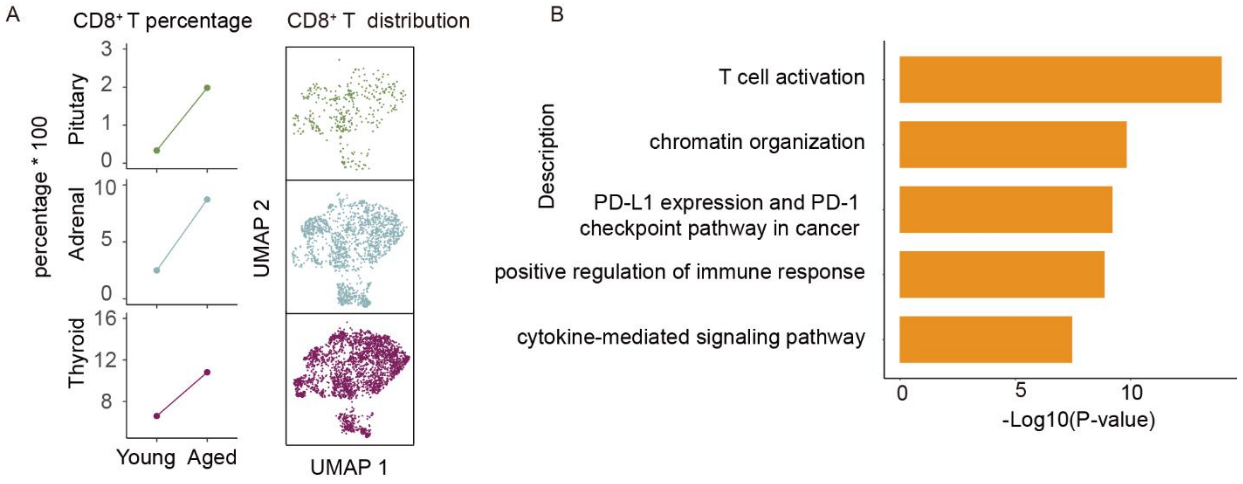

Figure S5 GZMK$^+$CD8$^+$ T cells expanded in multiple aged endocrine organs (related to Figure 4). (A) Line plots showing changes in the percentage of CD8$^+$ T cells in the indicated organs (left) and UMAP plots showing the distribution of CD8$^+$ T cells in the indicated organs (right). **(B)** The bar graph illustrating the upregulated pathways of GZMK$^+$CD8$^+$ T cells during aging.

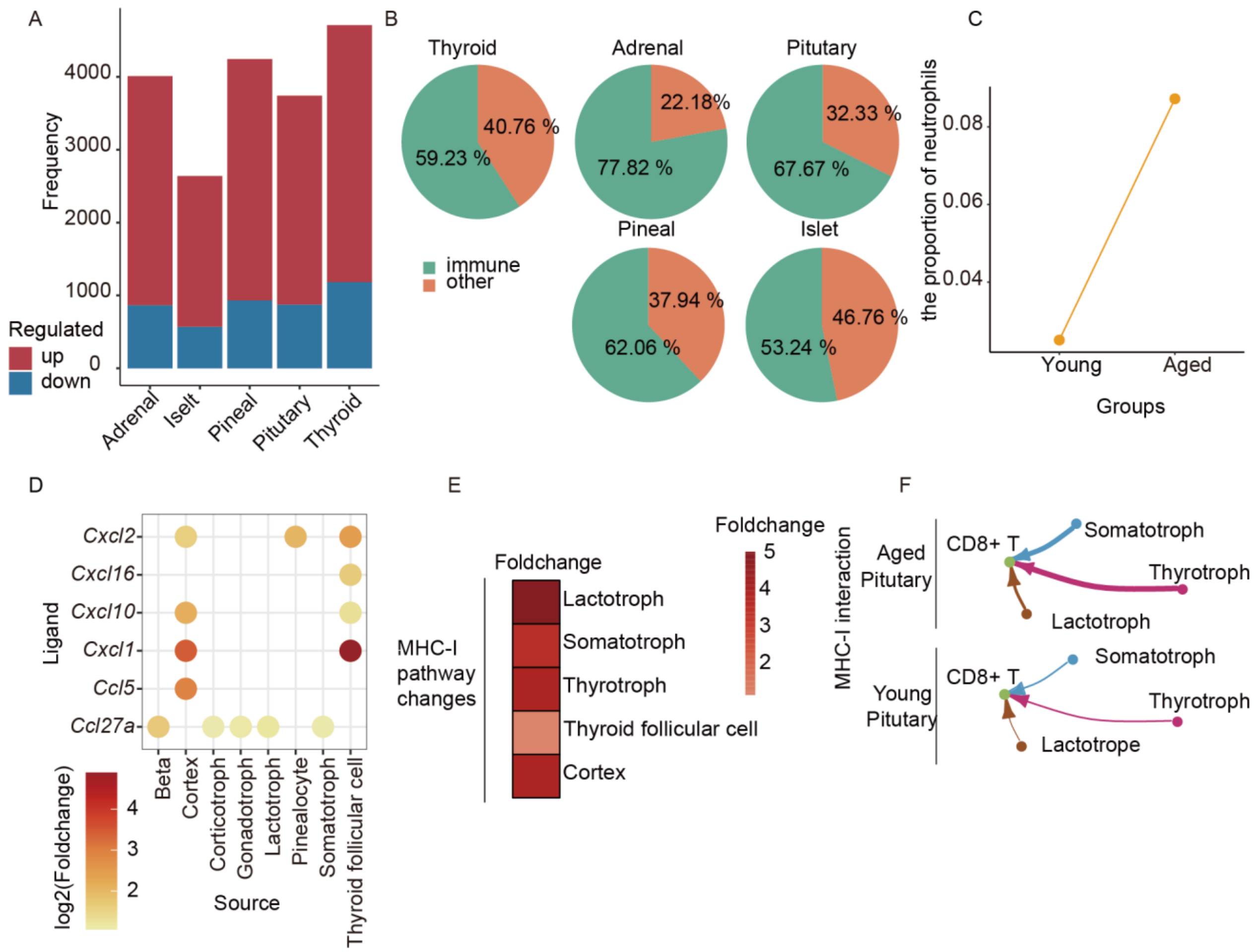

Figure S6 The immune microenvironments of endocrine organs are reshaped by aging (related to Figure 5). (A) Bar graphs showing the number of up- and downregulated ligand–receptor interactions in the indicated organs. **(B)** Pie charts illustrating the proportions of immune cell-associated interactions to all ligand–receptor interactions in the indicated organs. Immune cell-associated interactions are defined as interactions in which immune cells act as source or target cells. **(C)**The line plot showing the changes in the proportion of neutrophils during aging in the adrenal cortex. **(D)** The dot plot demonstrating changes in the expression of ligands related to the CCL and CXCL pathways during aging in the indicated functional endocrine cells. **(E)** The heatmap demonstrating changes in the communication probability of the MHC-I pathway between the indicated functional endocrine cell types and $CD8^+$ T cells. **(F)** Network plots showing the changes in the MHC-I pathway between the indicated functional endocrine cell types and $CD8^+$ T cells in young and aged pituitary glands.

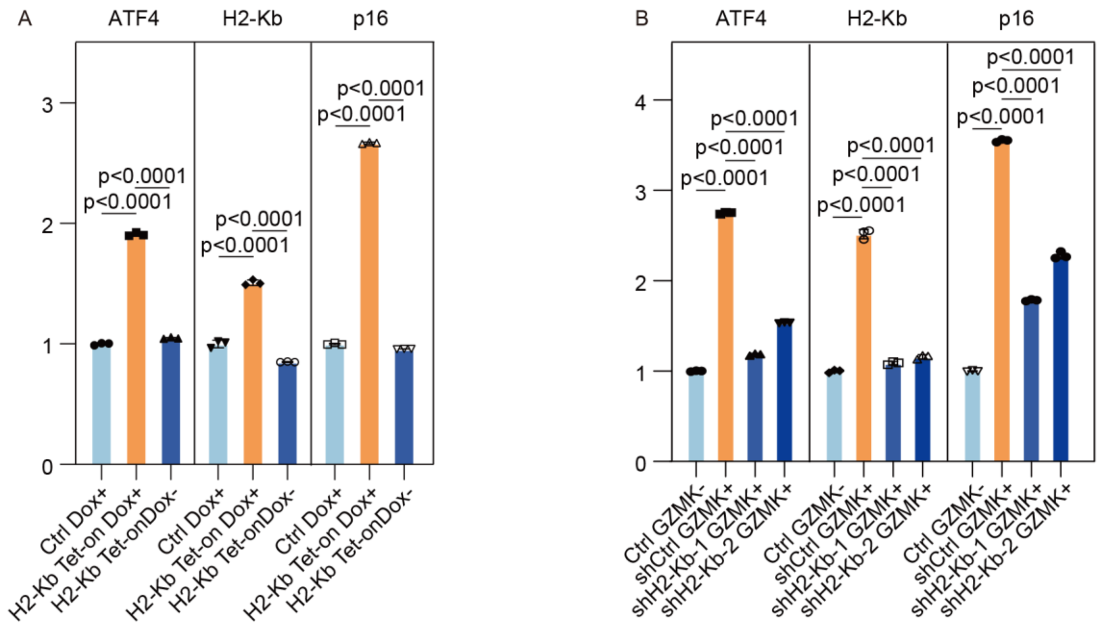

Figure S7 H2-Kb and GZMK regulates ATF4 and p16 expression in pituitary cells (related to Figure 6). (A) Western blot quantifications of the protein expression of H2-Kb, ATF4 and p16, following the overexpression of H2-Kb in pituitary cells. DOX, doxycycline. **(B)** Western blot quantifications of the protein expression of H2-Kb and ATF4 and p16 in pituitary cells treated with 100 ng/ml GZMK for knocking down H2-Kb expression and control group.

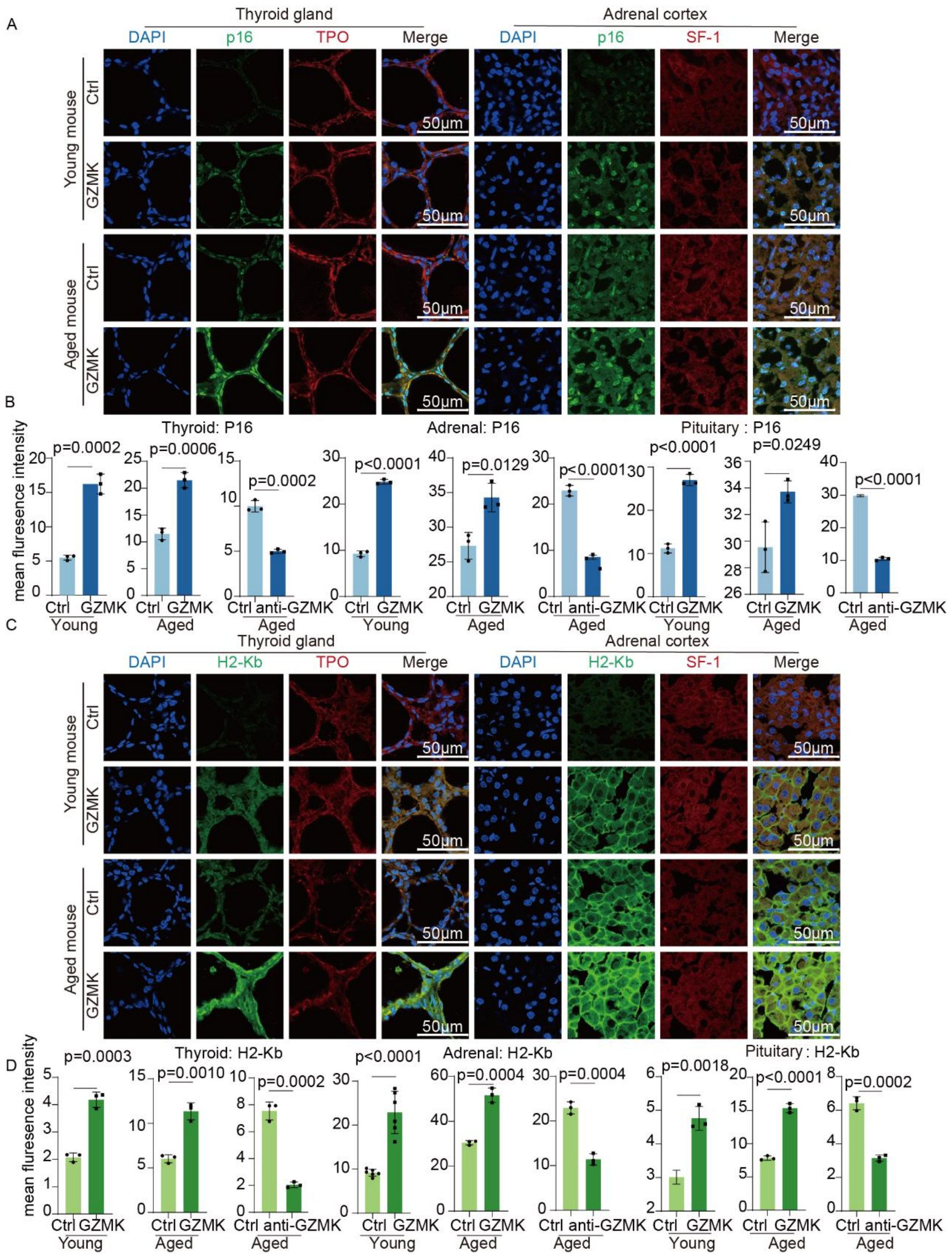

Figure S8 GZMK activates p16 and MHC-I (related to Figure 6). (A, C), Representative immunofluorescence images of p16 **(A)** and H2-Kb **(C)** coexpression with TPO (left) and SF-1 (right), respectively, across the indicated organs in the control and treatment groups (n = 3). **(B, D)** Quantification of TPO^{+}, SF-1^{+}, PIT1^{+} cells coexpressing p16 **(B)** and H2-Kb **(D)** across the indicated organs in the control and treatment groups (n = 3)

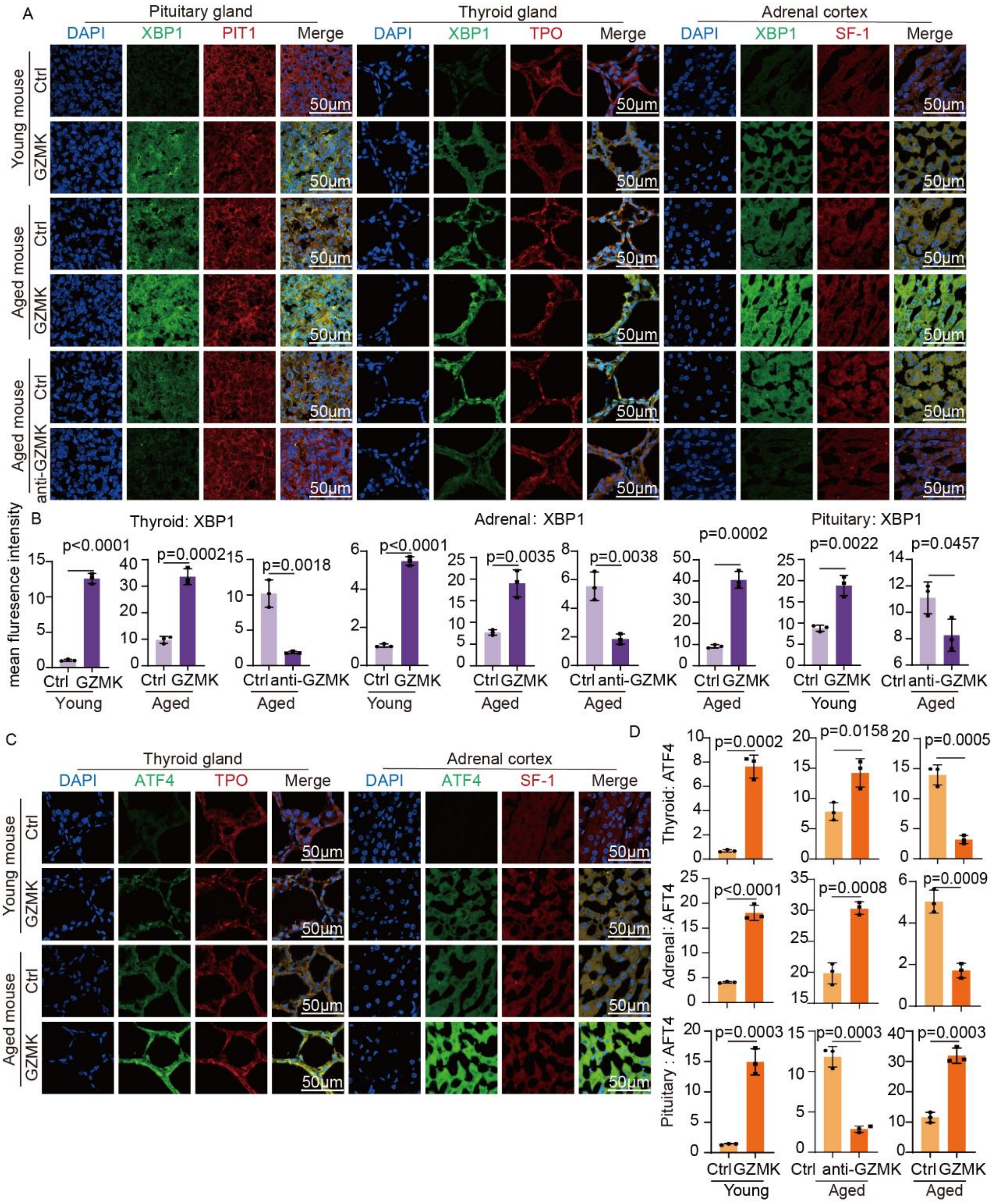

Figure S9 GZMK enhances the unfolded protein response (related to Figure 6). (A, B) Representative immunofluorescence images and quantification of XBP1 coexpression with PIT1, TPO and SF-1 across the indicated organs groups (n = 3). Ctrl, Control. **(C)** Representative immunofluorescence images of ATF4 coexpression with TPO (left) and SF-1 (right) across the indicated organs and groups (n = 3). **(D)** Quantification of ATF4 coexpression with TPO, SF-1 and PIT1 in the indicated groups.

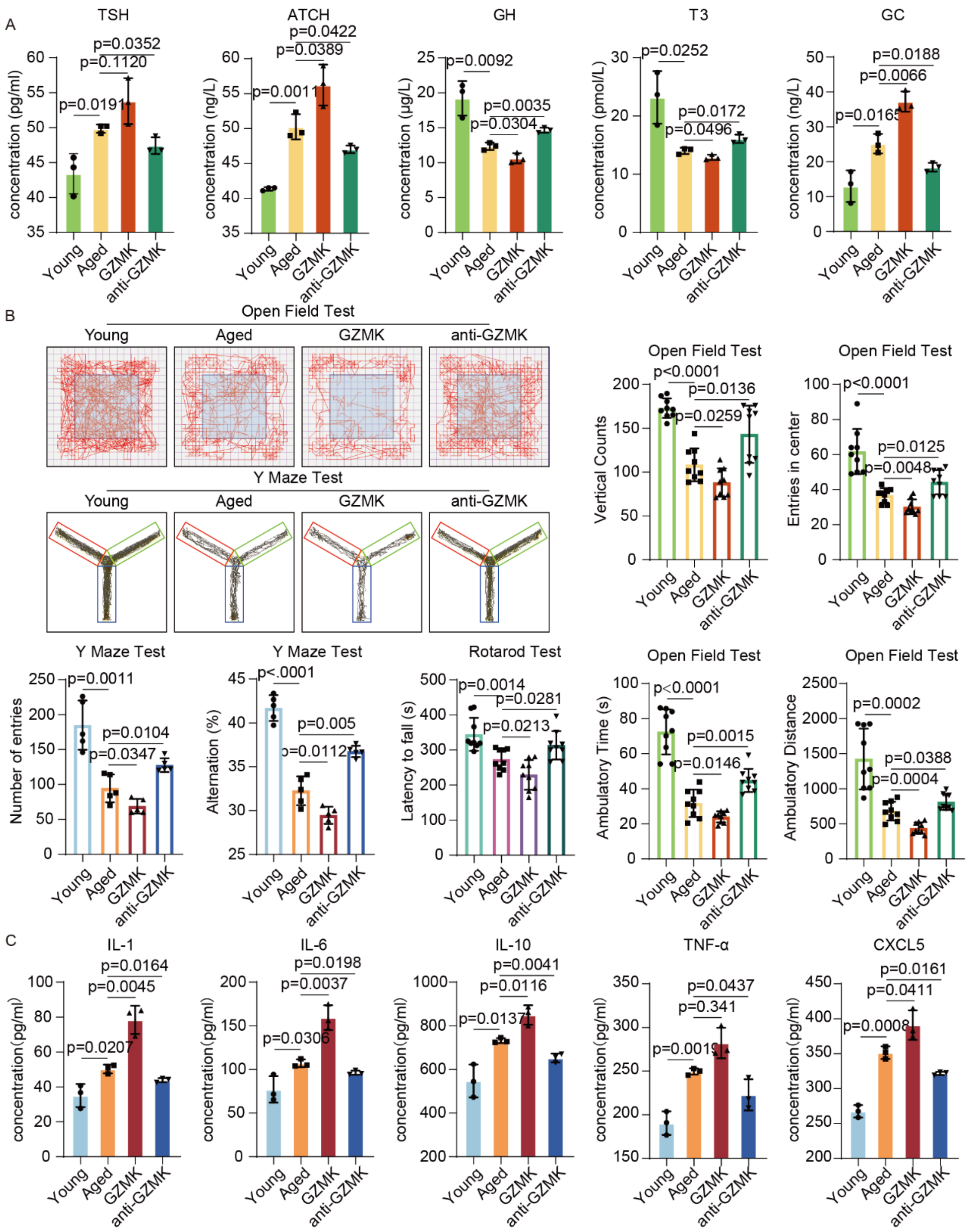

Figure S10 Anti-GZMK improves physiological indicators, behavioral phenotypes and hormone secretion capacity in aged mice. (related to Figure 6). (A) Levels of key endocrine hormones secreted by the pituitary gland, thyroid gland, and adrenal cortex, including TSH, ACTH, GH, T3, and GC, in the blood from the young, aged, GZMK treatment, and anti-GZMK treatment groups of mice (n = 3). **(B)** Key behavioral parameters and the travel tracks in the Y-maze test, rotarod test, and open-field test across the young, aged,

GZMK treatment, and anti-GZMK treatment groups of mice (n > 3). **(C)** Levels of SASP factors IL-1, IL-6, IL-10, TNF-α, and CXCL5 in the blood from the young, aged, GZMK treatment, and anti-GZMK treatment groups of mice (n = 3).

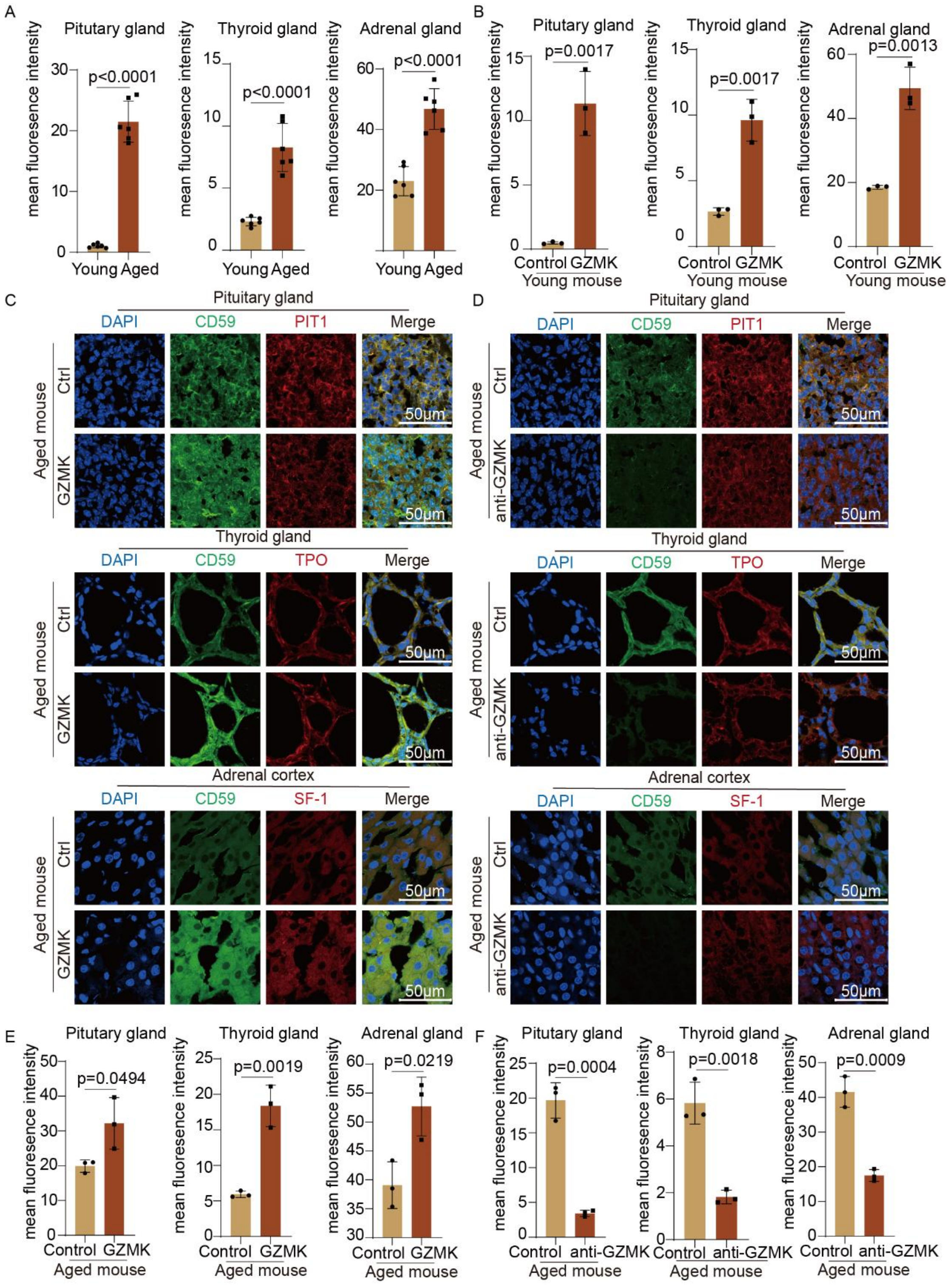
A
Pituitary gland
Thyroid gland
Adrenal gland
mean fluoresence intensity
p<0.0001
Young Aged
B
p=0.0017
p=0.0013
Control GZMK
Young mouse
C
Pituitary gland
DAPI CD59 PIT1 Merge
Aged mouse
Ctrl
GZMK
50μm
Thyroid gland
DAPI CD59 TPO Merge
Adrenal cortex
DAPI CD59 SF-1 Merge
D
anti-GZMK
E
p=0.0494
p=0.0019
p=0.0219
Aged mouse
F
p=0.0004
p=0.0018
p=0.0009
Control anti-GZMK

Figure S11 CD59 has an interplay with GZMK (related to Figure 7). (A) Quantification of CD59 coexpression with PIT1, TPO and SF-1 across the indicated organs in the young and aged groups (n = 6). **(B)** Quantificationsof CD59 coexpression with PIT1, TPO and SF-1 across the indicated organs and groups (n = 3). **(C, D)** Representative immunofluorescence images of CD59 coexpression with PIT1, TPO and SF-1 across the indicated groups and organs (n = 3). **(E, F)** Quantification of CD59 coexpression with PIT1, TPO and SF-1 in the indicated groups and organs.

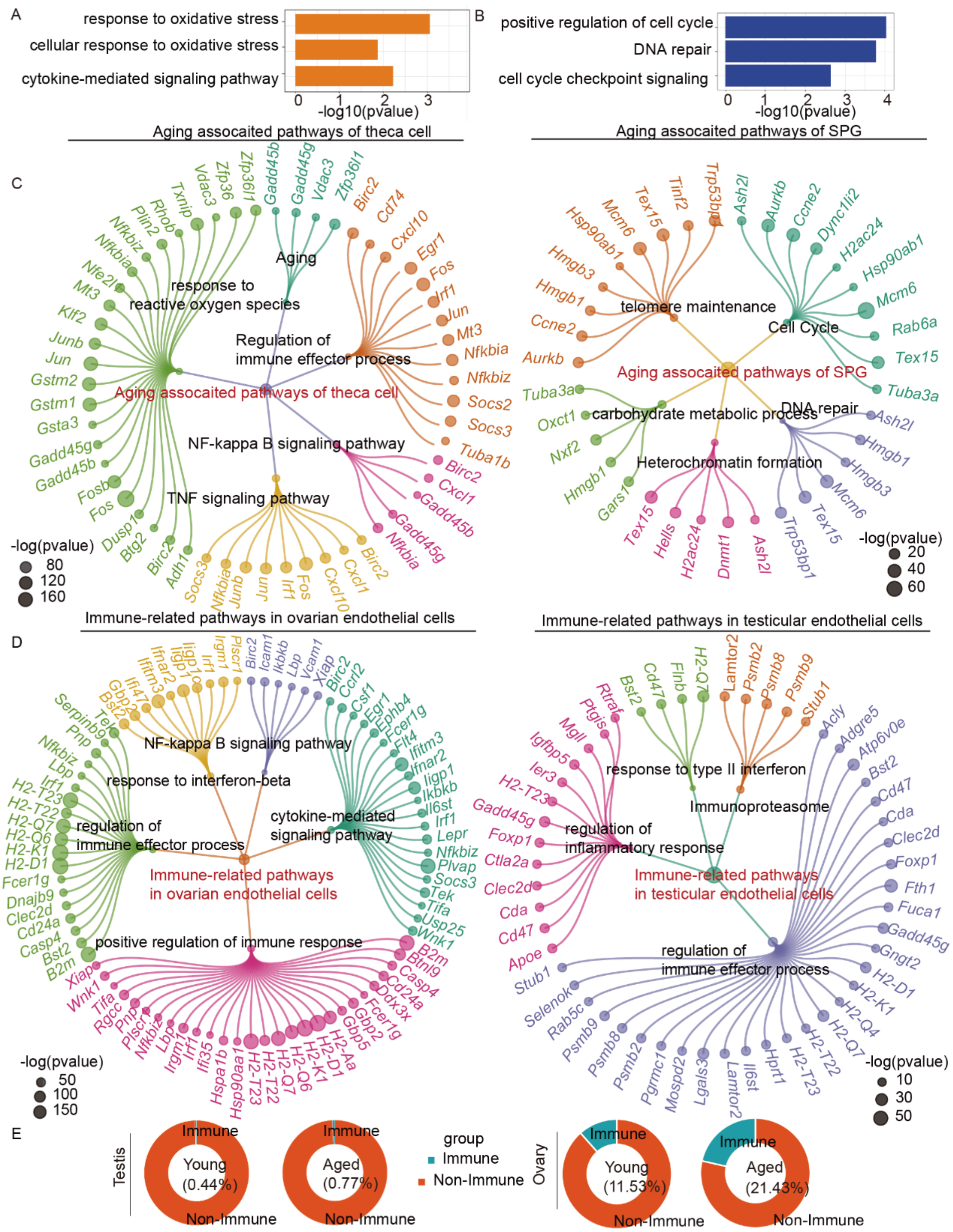

A
response to oxidative stress
cellular response to oxidative stress
cytokine-mediated signaling pathway
0 1 2 3
-log10(pvalue)
Aging assocaited pathways of theca cell
B
positive regulation of cell cycle
DNA repair
cell cycle checkpoint signaling
0 1 2 3 4
-log10(pvalue)
Aging assocaited pathways of SPG
C
Aging assocaited pathways of theca cell
Aging
response to reactive oxygen species
Regulation of immune effector process
NF-kappa B signaling pathway
TNF signaling pathway
-log(pvalue)
80
120
160
Aging assocaited pathways of SPG
telomere maintenance
Cell Cycle
carbohydrate metabolic process
DNA repair
Heterochromatin formation
-log(pvalue)
20
40
60
Immune-related pathways in ovarian endothelial cells
D
Immune-related pathways in ovarian endothelial cells
NF-kappa B signaling pathway
response to interferon-beta
cytokine-mediated signaling pathway
regulation of immune effector process
positive regulation of immune response
-log(pvalue)
50
100
150
Immune-related pathways in testicular endothelial cells
Immune-related pathways in testicular endothelial cells
response to type II interferon
Immunoproteasome
regulation of inflammatory response
regulation of immune effector process
-log(pvalue)
10
30
50
E
Testis
Immune
Young (0.44%)
Non-Immune
Immune
Aged (0.77%)
Non-Immune
group
Immune
Non-Immune
Ovary
Immune
Young (11.53%)
Non-Immune
Immune
Aged (21.43%)
Non-Immune

Figure S12 Aging reshaped the transcriptional landscape of the ovary and testis (related to Figure 8). (A) The bar plot showing upregulated representative aging pathways in the theca cells of the public ovary dataset. **(B)** The bar plot showing downregulated representative aging pathways in the SPGs of the public testis dataset. **(C)** Networks demonstrating commonly up-regulated DEGs and enriched pathways in theca cells (left), and commonly down-regulated DEGs and enriched pathways in SPGs (right). **(D)** Networks demonstrating immune-related common DEGs and corresponding enriched pathways in ovarian and testicular endothelial cells. (**E)** Pies chart showing the proportion of immune cells in the indicated group of public datasets.
